# Mineral medicines of the East: an analysis of records in historical Chinese and Japanese medical texts

**DOI:** 10.3389/fphar.2025.1584500

**Published:** 2025-09-22

**Authors:** Min Dai, Ding Wang

**Affiliations:** ^1^ Department of Japanese, School of Foreign Languages, Soochow University, Suzhou, China; ^2^ Department of Japanese, School of Foreign Languages, Hubei Minzu University, Enshi, China

**Keywords:** mineral medicine, East Asia, Chinese-Japanese historical medical texts, mercury-and mercury-compound-based mineral medicines, gypsum

## Abstract

**Background:**

Traditional Chinese medicine (TCM) in China and Kampo medicine in Japan are representative forms of traditional medicine in East Asia, in which mineral medicines constitute an indispensable source of therapeutic agents. Due to concerns about toxicity and safety, the frequency of mineral medicine use in modern times has declined compared with historical practice. Existing research on mineral medicines in traditional medicine has predominantly focused on toxicity and safety issues in an international context, overviews of historical mineral medicines in Japan, or records of specific mineral medicines in single ancient medical texts in China. However, there is a lack of comparative studies spanning multiple countries, historical periods, and a wide range of historical medical literature.

**Methods:**

This study utilized modern computational techniques and a self-constructed database, the Chinese–Japanese Traditional Medical Literature Corpus. Based on the medical history of China and Japan, 56 representative historical medical texts from the period 219–1863 were selected, from which data on mineral medicines were extracted. The methods of prescription metrology and data mining were applied to analyze the co-occurring medicines in prescriptions containing mineral medicines, while word frequency statistics were used to examine the conditions treated by these medicines. Data cleaning, statistical analysis, and visualization were performed using Microsoft Excel and Python scripts.

**Results:**

The “Prescription” category of historical medical texts is the primary source of mineral medicine data for both countries. A total of 106 mineral medicines were recorded in Chinese historical texts, compared with 100 in Japanese historical texts, with 97 mineral medicines shared between the two. Based on a cation-based classification system, the mineral medicines documented in the historical texts of China and Japan were divided into 16 categories; all 16 were found in Chinese texts, while Japanese texts contained 14 categories. The top three categories of mineral medicines by number of occurrences were the same in both countries, though their ranking order differed slightly. For pharmacological analysis, mercury- and mercury-compound-based mineral medicines (hereafter referred to as mercury-based mineral medicines) were selected due to their high toxicity and high number of occurrences. Historical Chinese texts recorded 189 medical conditions treated with mercury-based mineral medicines or compound prescriptions containing them, while Japanese texts recorded 98 such conditions, with two conditions unique to Japan. Six conditions were identified as core conditions strongly associated with mercury-based mineral medicines in both countries. Historical Chinese texts documented 257 co-occurring medicines with mercury-based mineral medicines, while Japanese texts recorded 240, with 17 species unique to Japan. Twelve co-occurring medicines were identified as core drugs strongly paired with mercury-based mineral medicines in both countries. Gypsum was selected for further pharmacological analysis, as it is included in both modern authoritative pharmacopoeias and ranks just below salt in number of occurrences in historical texts of both countries. Historical Chinese texts documented 429 co-occurring medicines with gypsum, while Japanese texts recorded 168. The core medicines strongly paired with gypsum showed minimal differences between the two countries. The top five conditions most strongly associated with gypsum, in terms of number of occurrences, were the same in both Chinese and Japanese historical texts, although their ranking varied slightly. Compared with the indications recorded in modern pharmacopoeias and medical literature of both countries, the descriptions of gypsum-related core conditions in historical texts were more diverse and detailed.

**Conclusion:**

The classification of historical medical texts in this study is based on the characteristics of their content. The types of historical texts serving as data sources for mineral medicines are similar in China and Japan, and the recorded mineral medicine species, compound types, and frequently recorded varieties also show a high degree of similarity, indicating that Kampo medicine in Japan extensively absorbed the theoretical foundations of mineral medicines from TCM. However, the higher number of occurrences of sodium compound–based mineral medicines in Japan, as well as the differences in the occurrence probabilities of commonly recorded mineral medicines between the two countries, to some extent reflect the localization tendencies of mineral medicine use in Kampo medicine. Mercury-based mineral medicines and gypsum documented in historical Chinese and Japanese medical texts showed minimal differences in associated conditions and co-occurring medicines. Many mercury-based mineral medicines shared generalizable features, highlighting the research significance and value of distinguishing mineral medicines by compound type to reveal overarching pharmacological trends. The comparison of gypsum’s principal therapeutic indications between historical and modern records revealed a clear trend toward a narrower application scope for mineral medicines in the modern era. From the perspective of preserving and inheriting traditional mineral medicine knowledge, a large amount of mineral medicine knowledge in historical Chinese and Japanese medical texts remains to be explored. Furthermore, research supported by objective data—such as analyses of the pharmacological effects of co-occurring medicines related to mineral medicines and studies on the associations between these co-occurring medicines and their related conditions—remains urgently needed.

## 1 Introduction

Despite the remarkable progress of modern scientific medicine, traditional medicine systems in East and Southeast Asia continue to serve as vital sources of knowledge for the discovery and development of novel therapeutics of natural origin (Brekhman and Grinevitch, 1981). Within East Asia, traditional Chinese medicine (TCM) in China and Kampo medicine in Japan share intertwined historical trajectories while each developing distinctive characteristics. Both countries possess a long-standing history of mineral medicines practices. In TCM, mineral medicines constitute one of the three major sources of traditional materia medica (Lin et al., 2025), with references to minerals such as cinnabar and realgar appearing as early as in the Classic of Mountains and Seas (Shan Hai Jing), representing some of the earliest documented medicinal uses of minerals (Liu et al., 2023).

Japan has a history of absorbing and adapting Chinese medicine, which gradually evolved into what is now known as Kampo medicine over the course of approximately 1,500 years (Kosoto, 1999). The use of mineral medicines in Japan can be traced back more than 1,300 years and holds a significant place in the country’s medical history. The earliest documented use of mineral medicines in Japan dates to the era of Emperor Shōmu (701–756). After his death in 756, various items he had used during his lifetime were stored and meticulously cataloged in the Shōsōin Repository of Tōdai-ji Temple in Nara. Among the medicinal substances recorded in the Shōsōin inventory, 21 were mineral medicines, accounting for approximately 30% of the total number of drugs listed (Masutomi, 1957).

In both modern China and Japan, mineral medicines have not been entirely abandoned; however, the range of substances currently used in clinical practice is quite limited. Among the 616 traditional Chinese medicines included in the Pharmacopoeia of the People’s Republic of China (2025 edition), issued by the National Medical Products Administration and effective from 1 October 2025, only 23 are mineral medicines (National Medical Products Administration of China, 2025). Similarly, in Japan, the Japanese Pharmacopoeia published by the Ministry of Health, Labour and Welfare in 2021 discusses in detail only three mineral medicines under the section on crude drugs (Ministry of Health and Labour and Welfare of Japan, 2021).

The significant disparity between the variety of mineral medicines used historically and those recognized in contemporary pharmacopeias in China and Japan suggests that the ancient use of mineral-based therapeutics remains a rich field for retrospective study and rediscovery. Ancient systems like Traditional Chinese Mineral Medicine (TCMM) provide more than historical footnotes—they offer clinically refined templates for modern metallodrug development (Bau et al., 2025). Historical medical texts from both China and Japan provide valuable historical data on the use of mineral medicines in antiquity and can serve as important resources for uncovering traditional pharmaceutical knowledge that may inform and inspire modern pharmacological advancements.

The documentation and study of mineral medicines in ancient medical texts have been particularly prominent in Chinese-language literature. Over the past decade, numerous studies have examined historical Chinese medical texts such as Shi Yao Er Ya, Bencao Tujing, Treatise on Cold Damage (Shang Han Lun), Synopsis of the Golden Chamber (Jin Kui Yao Lue), Xiaoer Yaozheng Zhijue, and Ming Yi Lei An. These investigations have explored ancient mineral medicines from multiple perspectives, including classification, toxicity and properties, clinical efficacy, frequency of use, dosage, geographic origin, and the role of cultural exchange (Chi et al., 2025; Su et al., 2025; Yan et al., 2022; Xiang and Zhang, 2021). In addition, a number of studies have focused on specific mineral medicines—such as potassium niter, succinum, gypsum, and halitum—analyzing their recorded names, variants, regions of origin, processing methods, therapeutic effects, medicinal properties, formulations, and comparisons between ancient and modern usage based on ancient Chinese texts (Tian et al., 2024; Chi et al., 2022; Liu et al., 2011).

In contrast, research written in Japanese has primarily provided broad overviews of ancient mineral medicines, with most studies published before the 21st century. These works focus on topics such as historical production sites of mineral medicines in Japan, records of their use in excavated wooden tablets, comparative documentation and applications of limonitum in ancient China and Japan, traditional modes of administration, and the mineral medicines preserved in the Shosoin Repository (Masutomi, 1957; Wada, 1999; Sugiyama, 1997; Tachiki, 1989; Watanabe, 2024; Monta, 2020). Despite being part of the shared East Asian traditional medical system, there has been no dedicated investigation into the documentation of mineral medicines in ancient Kampo medical texts. The extensive historical medical literature of Japan remains underexplored and holds considerable potential for further study.

In comparison, English-language literature places greater emphasis on the toxicity and safety concerns associated with mineral medicines in traditional medicine systems. For instance, one study evaluated the composition, physicochemical properties, and heavy metal toxicity of Armenian bole (Ab), a mineral medicine traditionally used in Iranian folk medicine since ancient times (Hosseinkhani et al., 2025). Another investigation examined the use and safety controversies of mineral- and metal-based medicines—particularly mercury—in the Ayurvedic system, analyzing them through the lens of heavy metal toxicity (Sikder, 2024). These studies reflect modern pharmacology’s cautious approach toward the use of traditional mineral medicines.

In addition to Iranian folk medicine (Hosseinkhani et al., 2025) and the Ayurvedic tradition (Sikder, 2024), similar concerns have been explored in other traditional medical systems such as Unani medicine (Makbul et al., 2018) and Afghan traditional medicine (Youno et al., 1991). Some English-language studies also explore the potential relationship between ancient mineral medicines and modern drug discovery. For example, the role of ancient iron–sulfur ([FeS]) clusters has been investigated in the search for new anticancer targets and therapies (Nghi et al., 2023). Arsenic trioxide (ATO)—now a frontline therapy for acute promyelocytic leukemia—traces its origins directly to arsenic sulfide formulations historically used in TCMM (Bau et al., 2025).

Unlike previous studies, the motivation of this research lies in conducting a cross-cultural and historical investigation of mineral medicines as recorded in historical medical texts from both China and Japan. Given the intrinsic connections between the traditional medical systems of the two countries, scholars have already provided detailed discussions on significant historical texts in the histories of TCM and Kampo medicine (Kosoto, 1999; Masutomi, 1957). Building upon these foundational studies, the present research aims to further explore the historical application of mineral medicines within the Chinese medical tradition, as well as how these practices were inherited and further developed within Japanese Kampo medicine.

## 2 Methods

### 2.1 Data source

The data used in this study are derived from a self-compiled corpus titled the Chinese–Japanese Traditional Medical Literature Corpus (Version 1.0). This corpus is currently under development and is based on 31 historical Chinese and Japanese medical texts (excluding veterinary works) included in the Dadi Corpus^1^ (Wang and Zong, 2024), with an additional 25 texts subsequently incorporated in the course of this study. As of now, the corpus comprises a total of 56 historical medical texts—32 from China and 24 from Japan.

**FIGURE 1:**
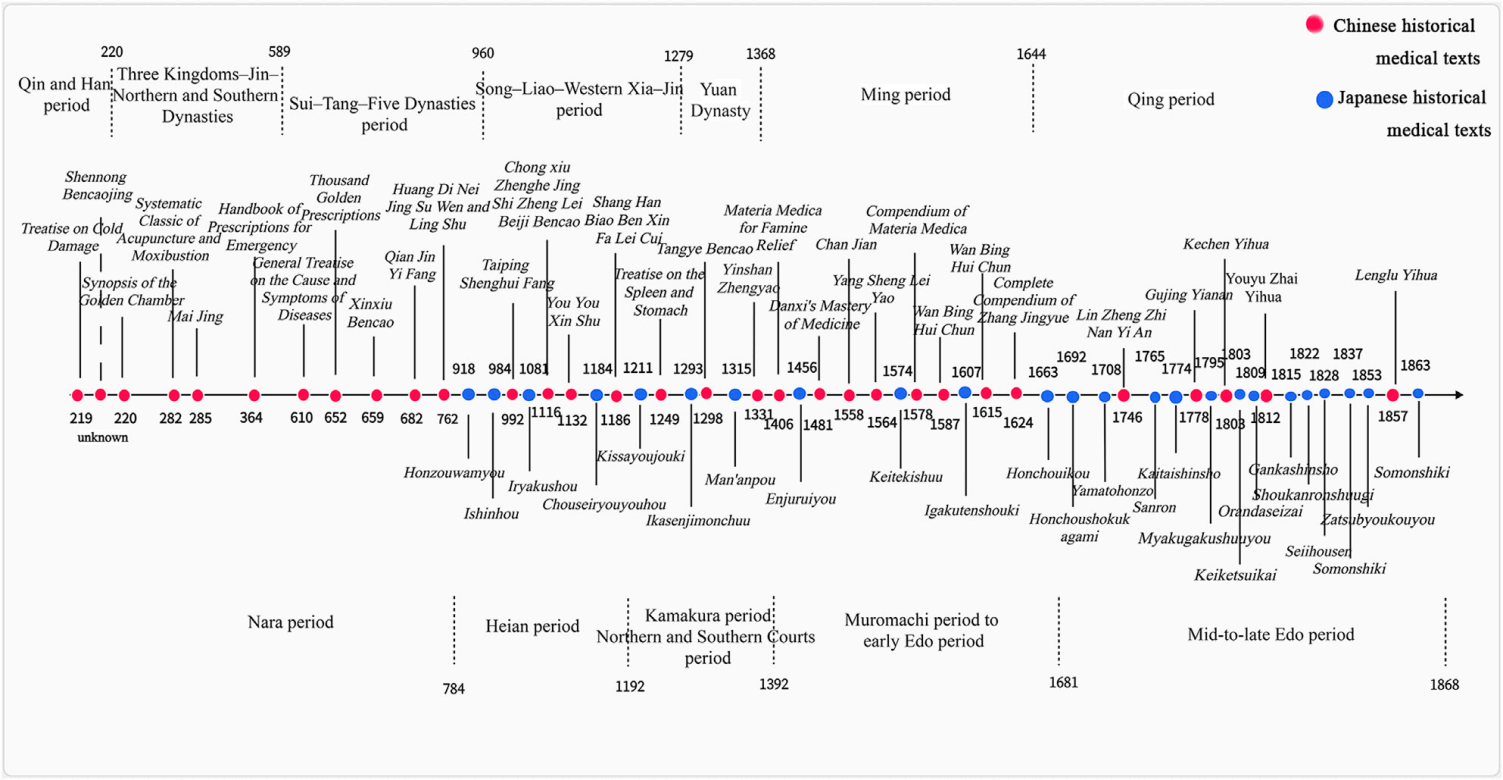
Timeline of 56 historical Chinese and Japanese medical texts.

The selection of texts in the Chinese–Japanese Traditional Medical Literature Corpus is grounded in the historical context of Sino-Japanese medical exchange, as well as the respective developmental trajectories of traditional medicine in both countries (Figure 1). During Japan’s Nara period, the implementation of the Taihō Code designated several Chinese medical classics—including the Mai Jing (Pulse Classic), the Systematic Classic of Acupuncture and Moxibustion (Jia Yi Jing), the Su Wen (Plain Questions), and the Ling Shu (Spiritual Pivot)—as official medical textbooks. These works, all composed prior to China’s Song dynasty, laid the foundational framework for the development of Kampo medicine in Japan.

In the subsequent Heian period, Japan began to compile its own medical texts. However, these early Japanese medical writings heavily relied on Chinese sources and had not yet formed a distinct medical theory of their own. For example, Japan’s oldest surviving medical text, Ishinho, as well as the second-oldest, Iryakusho, and the earliest Japanese materia medica dictionary, Honzouwamyou, all date to the Heian period and primarily draw upon Chinese medical knowledge.

During the Kamakura and Northern and Southern Courts periods, comprehensive Chinese medical encyclopedias from the Song dynasty—such as the Taiping Shenghui Fang (Formulary of the Taiping Era)—were transmitted to Japan. Influenced by these works, Japan produced its own extensive medical compilations that reflected the characteristics of the time, including the Man’anpo.

From the Muromachi period to the early Edo period, the medical philosophies of two of the “Four Great Masters” of the Jin-Yuan dynasties in China—Li Dongyuan and Zhu Danxi—gained significant influence in Japan. Representative Chinese medical texts from this era include Treatise on the Spleen and Stomach (Pi Wei Lun) by Li Dongyuan and Danxi’s Mastery of Medicine (Danxi Xinfa) by Zhu Danxi. In addition, Wan Bing Hui Chun by Gong Tingxian was widely reprinted in early Edo Japan and became a best-selling medical work (Mayanagi, 2014). The Compendium of Materia Medica (Bencao Gangmu) also played a crucial role in advancing Japanese herbal medicine and natural science during this period.In Japan, this era saw the emergence of influential physicians such as Manase Dosan, a leading figure of Gosei-ha School of Kampo, whose representative work was the Keitekishuu.

By the mid-to-late Edo period, various distinct medical schools had emerged in Japan, leading to the formal establishment of the Kampo medical system. Among these, the Koho-ha School of Kampo revered China’s Treatise on Cold Damage, while the Empirical Evidence School was influenced by the evidential scholarship movement of Qing China. The corpus includes works by prominent figures of the Empirical Evidence School, such as Taki Motoyasu, whose contributions include Shoukanronshuugi, Somonshiki, and Reisuushiki.

The renowned scholar of ancient medical texts in China, Ma Jixing (1990), classified ancient medical books into 12 categories: “Health Administration,” “Categories of Huang Di Nei Jing and Nan Jin,” “Zangxiang Theory,” “Etiology and Diagnostic Methods of TCM,” “Meridians and Acupuncture,” “Herbal and Other Medicinal Materials Category,” “Prescription,” “Zhang Zhongjing’s Prescriptions,” “Clinical Specialties Medical,” “Clinical Series,” “Works on External Treatment, Massage, Health Preservation and Zhuyou,” and “Others” (Ma, 1990). Following Ma’s classification framework, this study categorizes the 56 historical Chinese and Japanese medical texts used as data sources into 11 distinct types. The historical medical texts used as data sources in this study cover the majority of traditional text categories. A longitudinal perspective enables a comprehensive understanding of the historical use of mineral medicines in China and Japan, while a cross-sectional comparison allows for the examination of differences in how mineral medicines were recorded across various types of historical texts (see Table 1).

**TABLE 1:** Classification of historical medical texts from China and Japan.

本文分类	《黄帝内经》著作系统	《伤寒杂病论》系统	脏象、病源、诊法著作	医学方书	临床各科医书	本草学著作	针灸著作	养生著作	医史、医论、工具书著作	医案著作	西方医学著作
Text classification	Works System of “Huang Di Nei Jing”	Works System of “Treatise on Cold Damage and Synopsis of the Golden Chambe”	Viscera-State Theory/Etiology/Diagnostic Methods	Prescription	Clinical Specialties Medical	Works on Materia Medica (Pharmacology)	Works on Acupuncture and Moxibustion	Health Preservation	Medical History/Medical Discourses/Reference Works	Medical Case	Western Medical
China	黄帝内经素问 Huang Di Nei Jing Su Wen 黄帝内经灵枢 Huang Di Nei Jing Ling Shu	伤寒论 Treatise on Cold Damage 金匮要略 Synopsis of the Golden Chamber 伤寒标本心法类萃 Shang Han Biao Ben Xin Fa Lei Cui	诸病源候论 General Treatise on the Cause and Symptoms of Diseases 脉经 Mai Jing	太平圣惠方 Taiping Shenghui Fang 肘后备急方 Handbook of Prescriptions for Emergency 千金方 Thousand Golden Prescriptions 千金翼方 Qian Jin Yi Fang	幼幼新书 You You Xin Shu 产鉴 Chan Jian 丹溪心法 Danxi’s Mastery of Medicine 脾胃论 Treatise on the Spleen and Stomach 景岳全书 Complete Compendium of Zhang Jingyue 万病回春 Wan Bing Hui Chun 寿世保元 Shou Shi Bao Yuan	神农本草经 Shennong Bencaojing 新修本草 Xinxiu Bencao 本草纲目 Compendium of Materia Medica 救荒本草 Materia Medica for Famine Relief 政和新修经史证类备用本草 Chong xiu Zhenghe Jing Shi Zheng Lei Beiji Bencao 汤液本草 Tangye Bencao	针灸甲乙经 Systematic Classic of Acupuncture and Moxibustion	饮膳正要 Yinshan Zhengyao 养生类要 Yang Sheng Lei Yao	客尘医话 Kechen Yihua 友渔斋医话 Youyu Zhai Yihua 冷庐医话 Lenglu Yihua	临证指南医案 Lin Zheng Zhi Nan Yi An 古今医案按 Gujing Yianan	
Japan	霊枢識 (れいすうしき) Reisuushiki 素問識 (そもんしき) Somonshiki	傷寒論輯義 (しょうかんろんしゅうぎ) Shoukanronshuugi	脈学輯要 (みゃくがくしゅうよう) Myakugakushuuyou	医心方 (いしんほう) Ishinhou 医略抄 (いりゃくしょう) Iryakushou 万安方 (まんあんぽう) Man’anpou 啓迪集 (けいてきしゅう) Keitekishuu	産論 (さんろん) Sanron 雑病広要 (ざつびょうこうよう) Zatsubyoukouyou	本草和名 (ほんぞうわみょう) Honzouwamyou 本朝食鑑 (ほんちょうしょっかん) Honchoushokukagami 大和本草 (やまとほんぞう) Yamatohonzou	経穴彙解 (けいけついかい) Keiketsuikai	長生療養方 (ちょうせいりょうようほう) Chouseiryouyouhou 喫茶養生記 (きっさようじょうき) Kissayoujouki 延寿類要 (えんじゅるいよう) Enjuruiyou	医家千字文註 (いかせんじもんちゅう) Ikasenjimonchuu 本朝医考 (ほんちょういこう) Honchouikou	医学天正記 (いがくてんしょうき) Igakutenshouki	解体新書 (かいたいしんしょ) Kaitaishinsho 和蘭制剤 (おらんだせいざい) Orandaseizai 眼科新書 (がんかしんしょ) Gankashinsho 西医方選 (せいいほうせん Seiihousen

### 2.2 Data extraction and analysis

Due to the long developmental history of TCM, phenomena such as “different substances sharing the same name” and “different names referring to the same substance” are common (Wang, 2007). The Chinese Materia Medica (Zhonghua Bencao), which documents 1,762 medicinal substances, is the most comprehensive materia medica compiled to date, containing a large number of mineral medicines along with relatively detailed information (Huan et al., 2024).

To facilitate the rapid retrieval of mineral medicine information, 114 mineral medicine names (including official names, synonyms, and variant characters) across 16 categories recorded in the Chinese Materia Medica (National Administration of Traditional Chinese Medicine, 1999) were used as reference terms. These terms were individually searched within the corpus to generate a mineral medicine data table. The table includes the following information: mineral medicine name, official and variant names, recorded word forms (as found in Chinese and Japanese texts), year, work title, volume, example text passages where the mineral medicine appears, and the country of the medical text.

To gain a detailed understanding of the records and usage of mineral medicines in historical Chinese and Japanese medical texts, the mineral medicine dataset was manually cleaned in Excel. The data cleaning process followed several principles:

1. If the synonym of one mineral medicine is the standard name of another, the standard name was retained and the synonym removed. For example, “寒水石” (han shui shi) is both a synonym of gypsum and the standard name of crystalline mirabilite; in such cases, records in which “寒水石” represented gypsum were removed.
2. In cases where different mineral medicines share the same synonym and it was impossible to determine which specific mineral medicine was being referred to, all possible records were retained. For example, “黄石” (huang shi) is a synonym for both realgar and orpiment. When “黄石” appeared in a historical medical text without clearly indicating which of the two it referred to, both possibilities were kept.

Using Excel’s sorting and proportion calculation functions, statistics were generated on the species, occurrence counts, and proportions of mineral medicines recorded in historical Chinese and Japanese medical texts. Prescription metrology and data mining methods were applied to analyze the association rules between mineral medicines and other substances in prescriptions, enabling a comparison of similarities and differences in mineral medicine compatibility between China and Japan. Data visualization was performed using Python scripts, and online chart-generation platforms, providing a more intuitive and efficient understanding of the overall characteristics and distinctive features of mineral medicine records in historical Chinese and Japanese medical texts.

## 3 Results

### 3.1 Overview and comparative summary of mineral medicines in historical Chinese and Japanese medical texts

**FIGURE 2:**
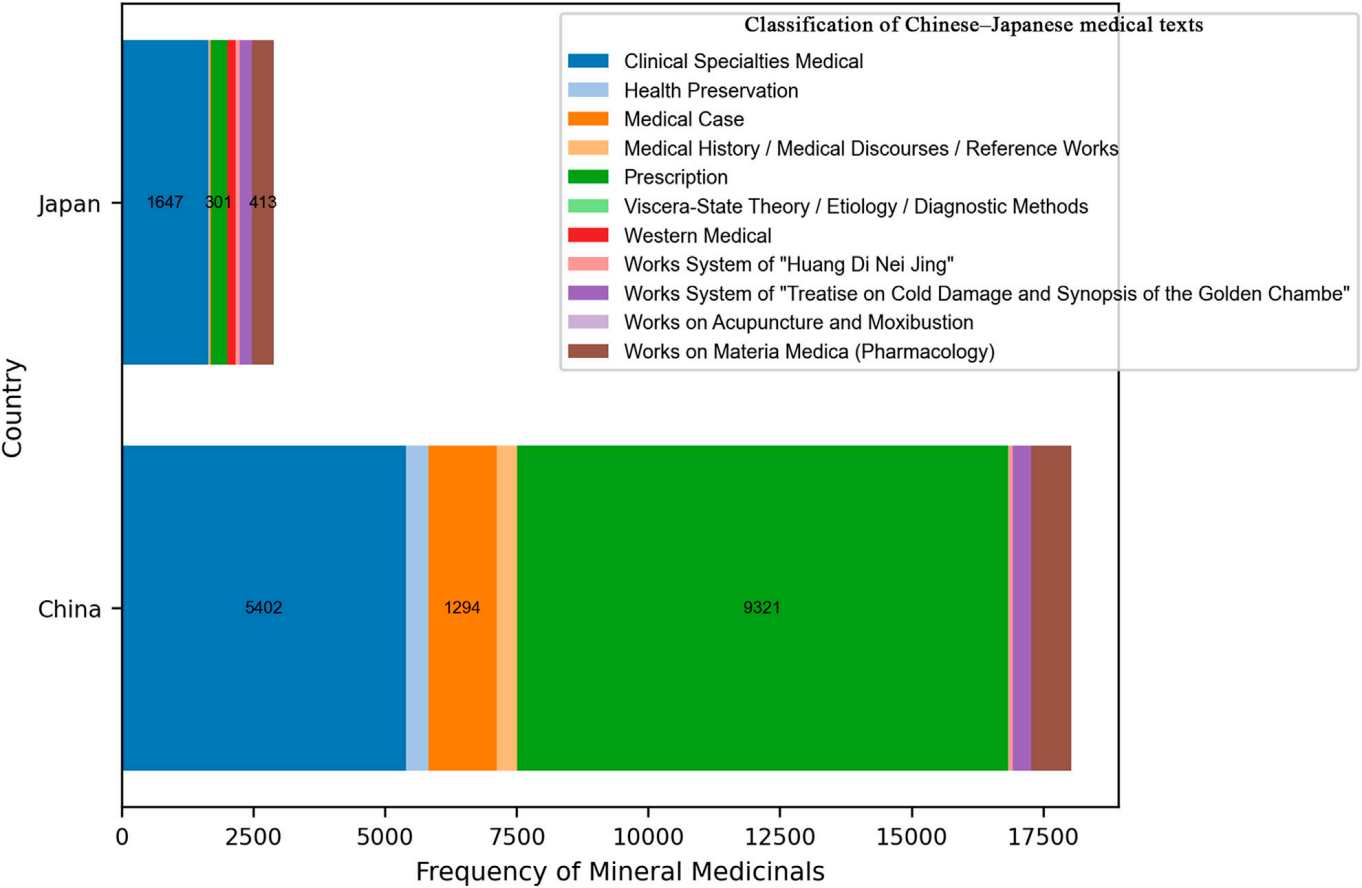
Classification of Chinese–Japanese medical texts.

Among the 32 historical Chinese medical texts included in the mineral medicine data table, 30 contain records of mineral medicines—with the exceptions of Mai Jing (Pulse Classic) and the Systematic Classic of Acupuncture and Moxibustion. All 24 Japanese texts included in this study contain references to mineral medicines. In the Chinese texts, mineral medicines are most frequently found in the categories of Prescription, Clinical Specialties, and Medical Case Records. In contrast, in the Japanese texts, they primarily appear in Clinical Specialties, Works on Materia Medica (Pharmacology), and Prescription. For both countries, the Prescription categories serve as the primary sources for the statistical data on mineral medicines (see Figure 2).

A total of 109 distinct mineral medicines were identified across the historical Chinese and Japanese medical texts included in this study, with 20,922 occurrences in total. Among these, 106 types were found in the Chinese texts, accounting for 18,033 instances, while 100 types appeared in the Japanese texts, totaling 2,889 instances. There were 97 mineral medicines common to both Chinese and Japanese texts, representing 94% of the total number recorded in the Chinese texts and 97% of those in the Japanese texts (see Table 2). Among the mineral medicines recorded in the Japanese texts, 47 have been identified as historically produced in Japan, based on evidence found in both historical Japanese medical texts and other related historical documents. These are highlighted in gray in Table 2 (Fukae, 2024; Tamba, 2024; Onda, 1901; Fujikawa, 1944; Tanaka, 1988; Tanaka, 1901; Tokoi and Saito, 1979; Okamoto, 1944; Ihara, 1901).

**TABLE 2:** Comparative frequency of mineral medicines in historical Chinese and Japanese medical texts.

Mineral Medicines	English Names	Counts (China)	Counts (Japan)	Total	Mineral Medicines	English Names	Counts (China)	Counts (Japan)	Total
食盐	Salt	1701	531	2232	曾青	Azurite	52	9	61
石膏	Gypsum	1642	392	2034	温泉	Hot Spring	32	27	59
朱砂	Cinnabaris	1476	188	1664	铁落	Iron Chippings	37	20	57
雄黄	Realgar	1012	71	1083	石碱	Alkali	48	7	55
滑石	Talcum	777	95	872	针砂	Zhensha (needle iron ore)	35	15	50
白矾	Alumen	796	58	854	灵砂	Cinnabaris Artificiali	16	32	48
龙骨	Dragon’s Bones	667	71	738	空青	Azurite (globular or hollow)	39	7	46
硫黄	Sulfur	620	58	678	黄矾	Fibrofessite	39	1	40
铁	Ferrum	545	105	650	铁精	Ashes in Iron-smelting Furnace	35	4	39
轻粉	Calomelas	549	38	587	理石	Fibrous Gypsum	27	10	37
水银	Mercury	518	53	571	自然铜	Pyritum	35	2	37
芒消	Mirabilite	483	78	561	玄精石	Selenite	31	5	36
铅丹	Red Lead	484	33	517	花蕊石	Ophicalcitum	24	8	32
琥珀	Succinum	389	79	468	蛇含石	Shehanshi	22	10	32
铅粉	Lead-powder	388	30	418	地浆	Dijiang	21	10	31
赤石脂	Halloysitum Rubrum	293	49	342	卤碱	Salt Alkali	22	8	30
石炭	Coal	263	53	316	粉霜	Chloride Mercure	28	2	30
磁石	Magnetitum	259	24	283	炉甘石	Calamina	29	0	29
铅	Galenite	207	58	265	朴消	Natrii Sulfas	14	12	26
硼砂	Borax	241	9	250	白垩	Chalk	19	7	26
消石	Niter	177	66	243	银朱	Artificial Mercuric Sulphide	24	2	26
寒水石	Crystalline Mirabilite	199	26	225	泉水	Spring	18	7	25
大青盐	Halitum	193	16	209	石燕	Fossil Shell of Spirifer	22	3	25
伏龙肝	Furnace Soil	180	17	197	绿盐	Atacamite	25	0	25
密陀僧	Lithargite	176	11	187	不灰木	Asbestos	22	1	23
龙齿	Dens Draconis	177	9	186	硇砂	Sal Ammoniac	16	6	22
钟乳石	Stalactitum	166	17	183	赤铜屑	Red Coppe	20	2	22
禹余粮	Limonitum	144	37	181	长石	Anhydrite	12	8	20
扁青	Blue Malachite	161	11	172	方解石	Calcite	12	5	17
石灰	Lime-stone	146	23	169	乳花	Stalactite Flower	13	3	16
砒霜	Arsenic	157	8	165	铁锈	Ferrum Oxydatum	12	3	15
金箔	Aurun Foil	146	14	160	红粉	Hydrargyri Oxydum Rubrum	12	2	14
紫石英	Fluoritum	150	8	158	麦饭石	Maifanitum	14	0	14
胆矾	Chalcanthite	140	15	155	孔公孽	Kong Gong Nie	9	4	13
雌黄	Orpiment	129	10	139	井底泥	Well Bottom Mud	10	2	12
铁粉	Iron-powder	124	13	137	姜石	Ginger Stone	10	2	12
铅霜	Lead-cream	117	1	118	铁浆	Iron-plasm	8	4	12
锡	Stannum	96	13	109	殷孽	Impregnated Calcite	8	3	11
白石英	Quartz	98	5	103	无名异	Pyrolusite	10	0	10
秋石	Qiushi	94	4	98	石脑油	Petroleum	8	2	10
云母	Mica	68	23	91	鹅管石	Coral Skeleton	10	0	10
绿矾	Melanteritum	77	14	91	黄石脂	Hydromica	6	4	10
代赭石	Haematitum	57	29	86	玛瑙	Agate	6	2	8
阳起石	Tremoliteor Tremoliteasbestos	71	11	82	铁华粉	Rust Powder is Made of Iron and Acetic Acid	3	3	6
青礞石	Lapis Chloriti	61	17	78	光明盐	Bright Salt	3	2	5
玄明粉	Weathered Sodium Sulfate	68	6	74	石床	Stalactite	2	3	5
礜石	Arsenopyrite	31	43	74	铅灰	Lead Ash	2	3	5
玉	Jade	56	13	69	咸秋石	Prepared Salt	3	0	3
白石脂	Halloysitum Album	61	8	69	甘土	Montmorillonite	0	3	3
砒石	Arsenic Sublimate	61	5	66	紫硇砂	Halite Violaceous	2	0	2
铜绿	Verdigris	63	3	66	绿青	Malachite	1	1	2
冰	Ice	37	27	64	金礞石	Lapis Micae Aureus	0	2	2
浮石	Pumice	58	6	64	石蟹	Fossilia Brachyura	0	1	1
银箔	Argentum Foil	54	8	62	红升丹	Hong-Shengdan	1	0	1
					金精石	Vermiculite	1	0	1

The types of mineral medicines recorded in historical Chinese and Japanese medical texts show a high degree of similarity, and the primary genres of texts in which these substances appear are also largely consistent between the two countries. This suggests that traditional Japanese medicine extensively absorbed the theoretical foundations of mineral medicine from TCM, and that the textual patterns through which such knowledge was transmitted in TCM were also adopted and applied within Kampo medical literature.

In addition to records of mineral production in Japan, historical sources such as A History of Diseases in Japan document the clinical use of mineral medicines in premodern times. These include the application of realgar to the nasal cavity for epidemic prevention; the combination of realgar and orpiment with herbal medicines to prevent cholera; the use of mirabilite as a purgative for cholera; the use of mirabilite and niter to treat typhoid fever; gypsum to relieve the heat and thirst symptoms associated with typhoid fever; and various treatments for dysentery such as purgation with natrii sulfas, astringency with halloysitum rubrum, external application of roasted salt to the anus, and salt-wine enemas (Fujikawa, 1944). These records suggest that mineral medicines, introduced from China as part of transmitted pharmaceutical knowledge, were not only documented in texts but also deeply embedded in clinical practice in historical Japanese medicine.

#### 3.1.1 Cation-based comparison of mineral medicines in China and Japan

**FIGURE 3:**
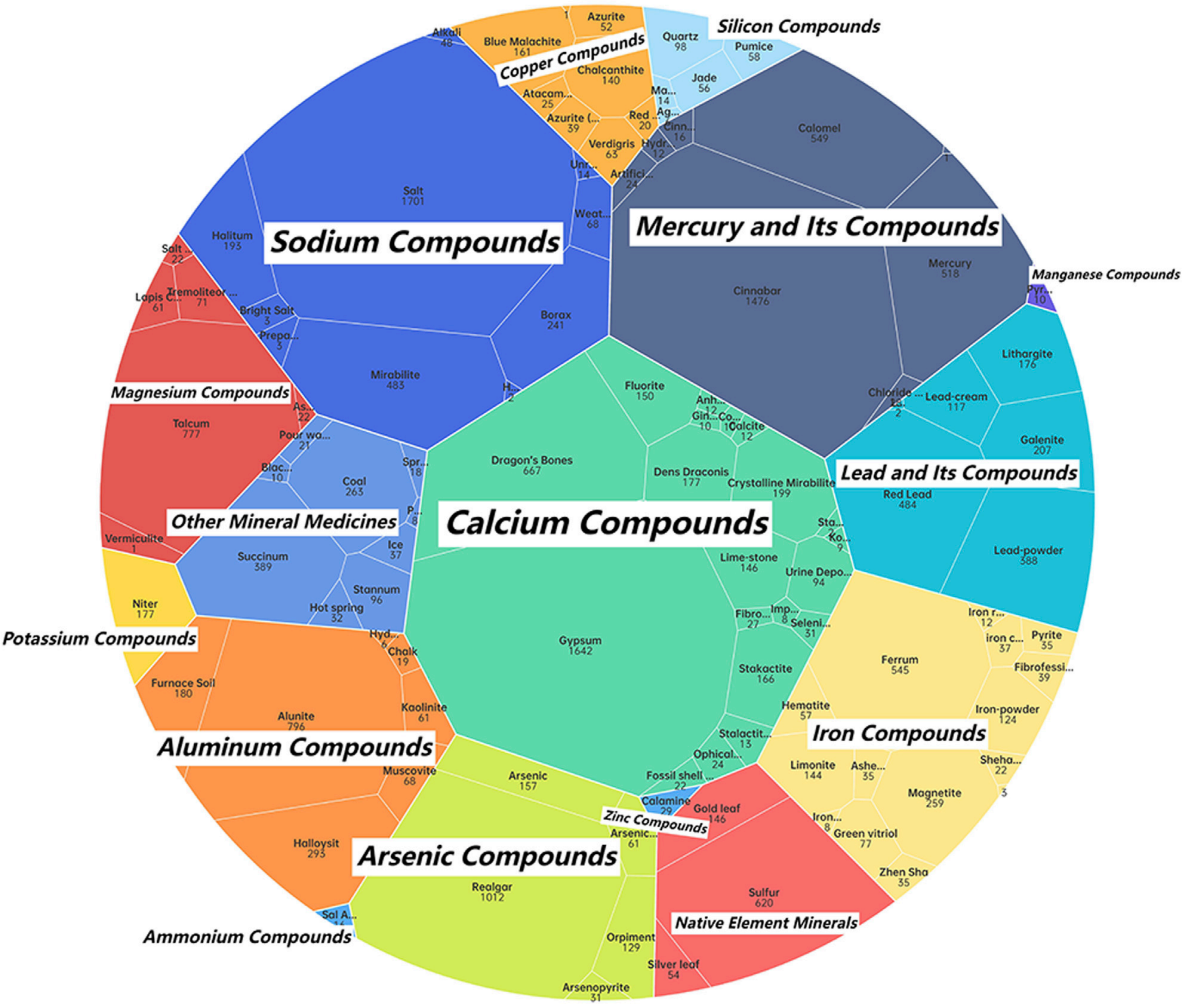
Classification of mineral medicines in historical Chinese medical texts.

**FIGURE 4:**
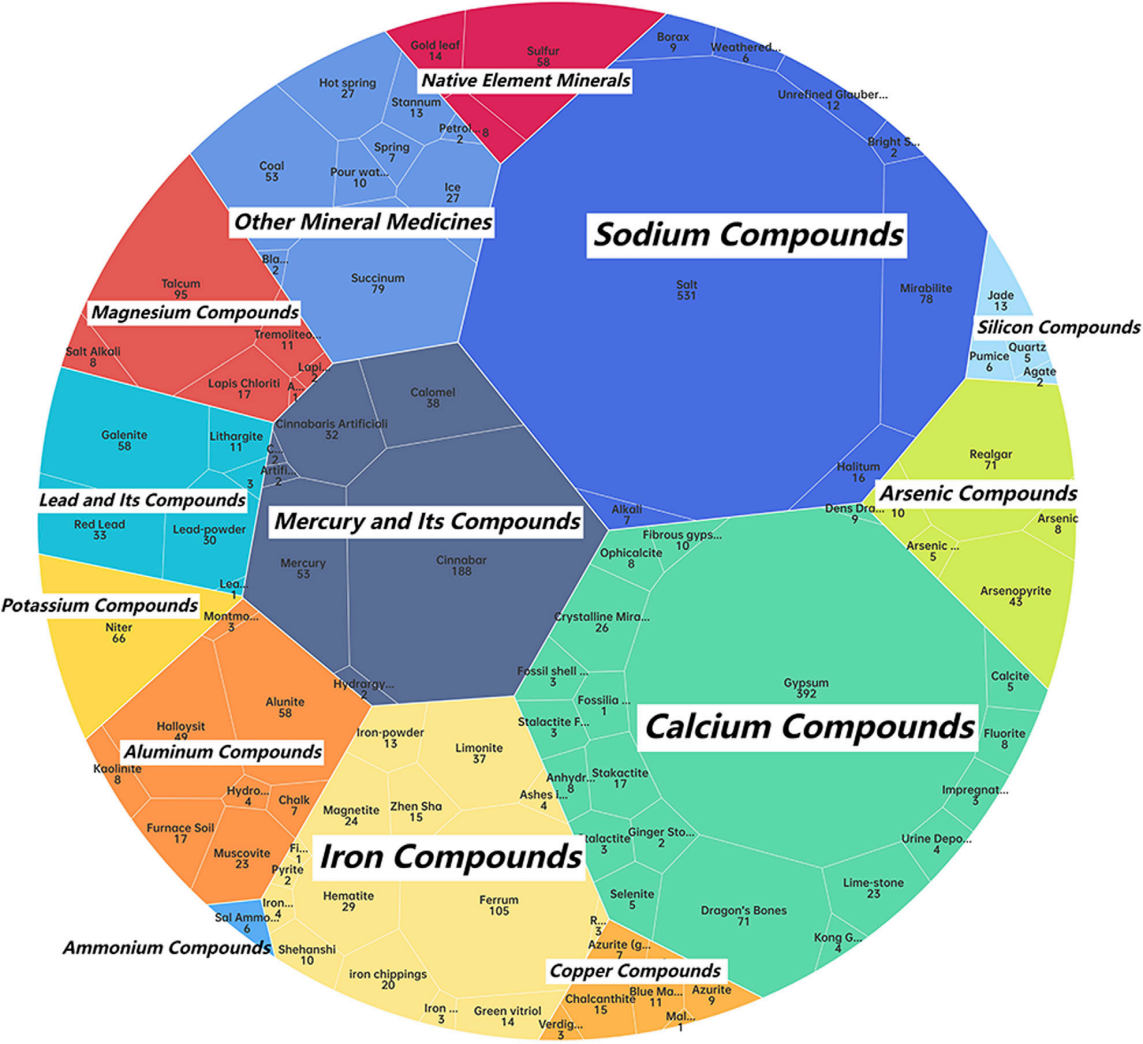
Classification of mineral medicines in historical Japanese medical texts.

The classification of mineral medicines constitutes an important aspect of theoretical research on mineral medicines, and the methods of classification have varied throughout history (Lin et al., 2025). It is generally believed that cations play a more significant role in determining the pharmacological effects of mineral medicines; therefore, works such as Chinese Materia Medica and Zhong Yao Da Ci Dian classify mineral medicines according to their cation types (Lin et al., 2025). Drawing on the cation-based classification system in Chinese Materia Medica (National Administration of Traditional Chinese Medicine, 1999), this study categorizes the mineral medicines recorded in historical Chinese and Japanese medical texts into 16 compound types, and, based on their frequency of occurrence, calculates the number of occurrences of each type in the two corpora, as illustrated in Figures 3, 4.

According to the statistical analysis, a total of 16 categories of mineral compounds were identified in the historical Chinese medical texts, while 14 categories were found in the historical Japanese medical texts. In the Chinese texts, the top three categories with the highest number of occurrences were: Calcium Compounds (e.g., gypsum, dragon’s bones), Sodium Compounds (e.g., salt, mirabilite), Mercury and Its Compounds (e.g., cinnabar, calomel). In the Japanese texts, the top three categories with the highest number of occurrences were: Sodium Compounds (e.g., salt, mirabilite), Calcium Compounds (e.g., gypsum, crystalline mirabilite), Mercury and Its Compounds (e.g., cinnabar, mercury)^2^.

From the perspective of mineral medicine composition, the historical records of China and Japan show little difference. Japan lacks mineral medicines of the zinc compound and manganese compound categories, both of which account for a small proportion of occurrences in Chinese historical medical texts, indicating that these categories were also infrequently used in China’s history. The zinc compound mineral medicine recorded in Chinese historical texts is calamina (ludanshi). According to the research of Japanese scholar Masutomi K, calamina was used in ophthalmology during China’s Ming Dynasty and was later introduced into Japan for the same purpose, suggesting that it was a relatively late-introduced mineral medicine in historical terms (Masutomi et al., 1953). The absence of this category of mineral medicines from Japanese historical medical texts within the scope of this study may be due to its limited historical use in both countries and its relatively short period of appearance as a mineral medicine.

In comparison between China and Japan, the category with the highest number of occurrences in Japanese historical records is sodium compounds, represented by salt and mirabilite, whereas in China it is calcium compounds, represented by gypsum and dragon’s bones. This indicates a certain difference in preference for the chemical composition of mineral medicines between the two traditional medical systems. In addition, mercury-based mineral medicines—known for their high toxicity—rank high in number of occurrences in the historical records of both countries. Analyzing the application records of mercury-based mineral medicines in these ancient texts offers insights into how Chinese and Japanese traditional medicine historically approached the use of toxic mineral medicines.

#### 3.1.2 Cation-based comparison of mineral medicines in China and Japan

**FIGURE 5:**
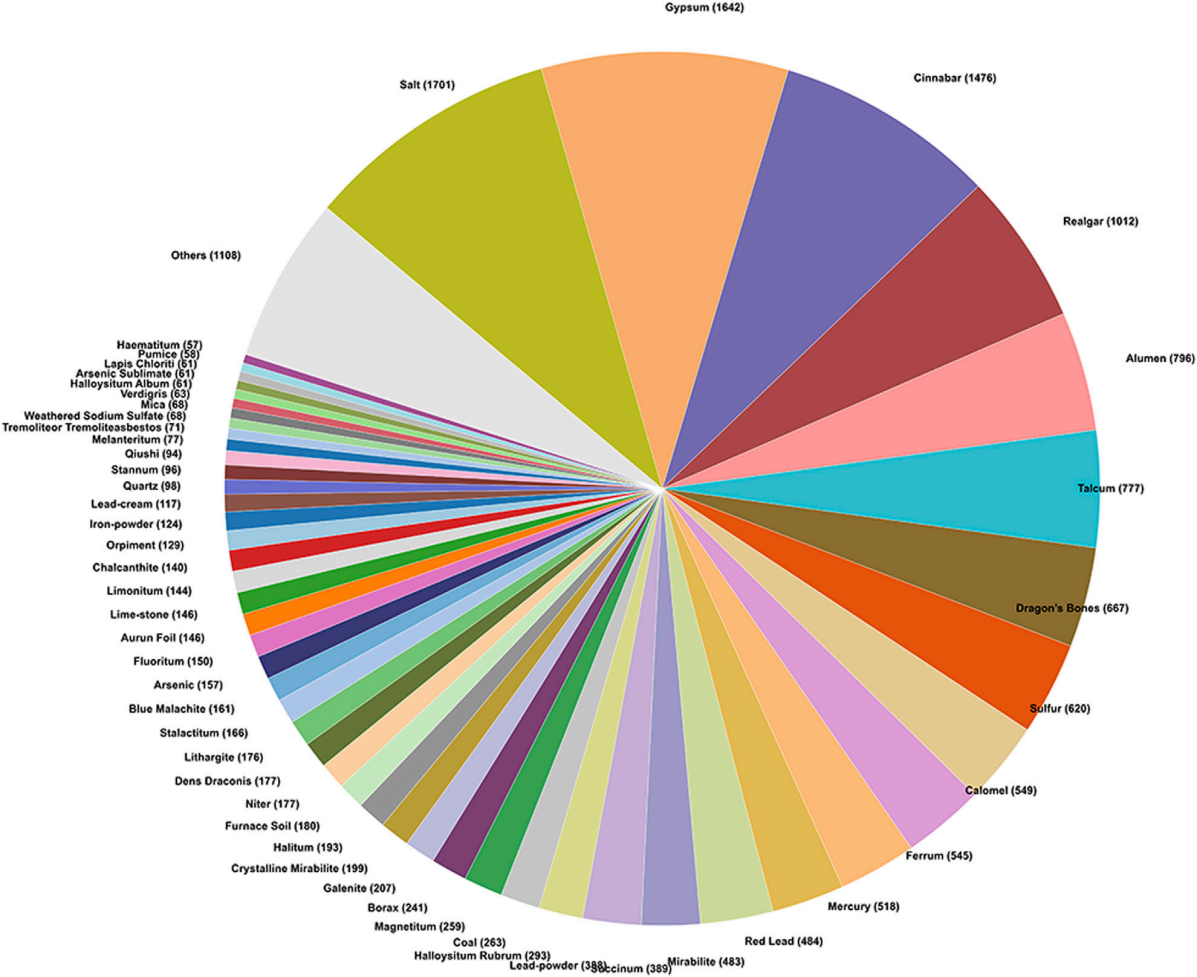
Proportion of occurrences of mineral medicines in historical Chinese medical texts (top 50 shown individually; all others grouped as “others”).

**FIGURE 6:**
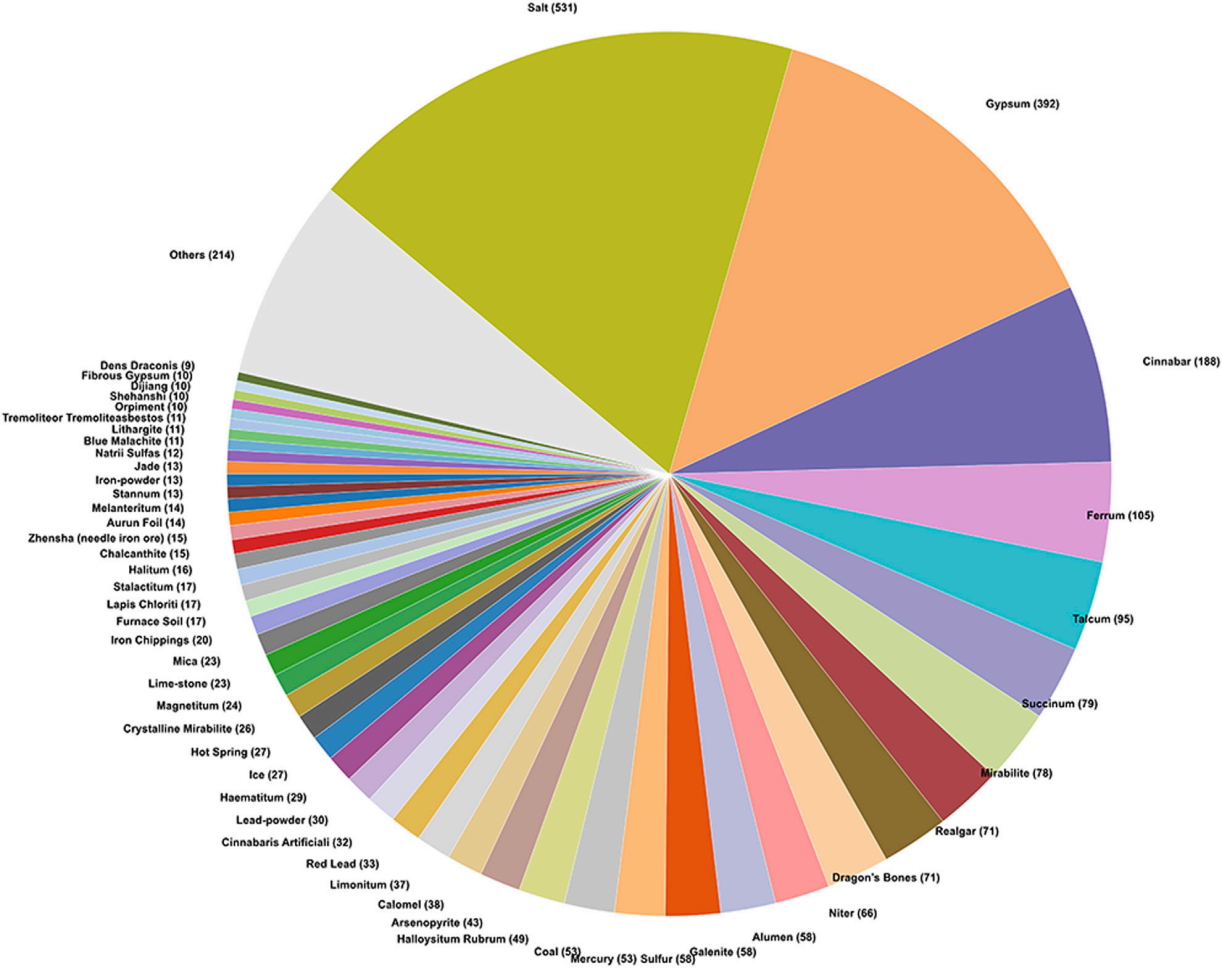
Proportion of occurrences of mineral medicines in historical Japanese medical texts (top 50 shown individually; all others grouped as “others”).

Building on the cation-based classification of mineral medicines, the preceding comparison between Chinese and Japanese historical medical texts has revealed certain characteristic patterns. To further elucidate these findings, it is essential to examine which mineral medicines were most frequently recorded, how their occurrence frequencies varied, and whether the commonly recorded mineral medicines in both traditions exhibit notable similarities. Accordingly, this study identifies the top ten most frequently recorded mineral medicines in the historical medical texts of each country and conducts a comparative analysis. This approach allows for a more fine-grained understanding of the representation and prominence of mineral medicines in the traditional medical histories of China and Japan (Figures 5, 6).

**FIGURE 7:**
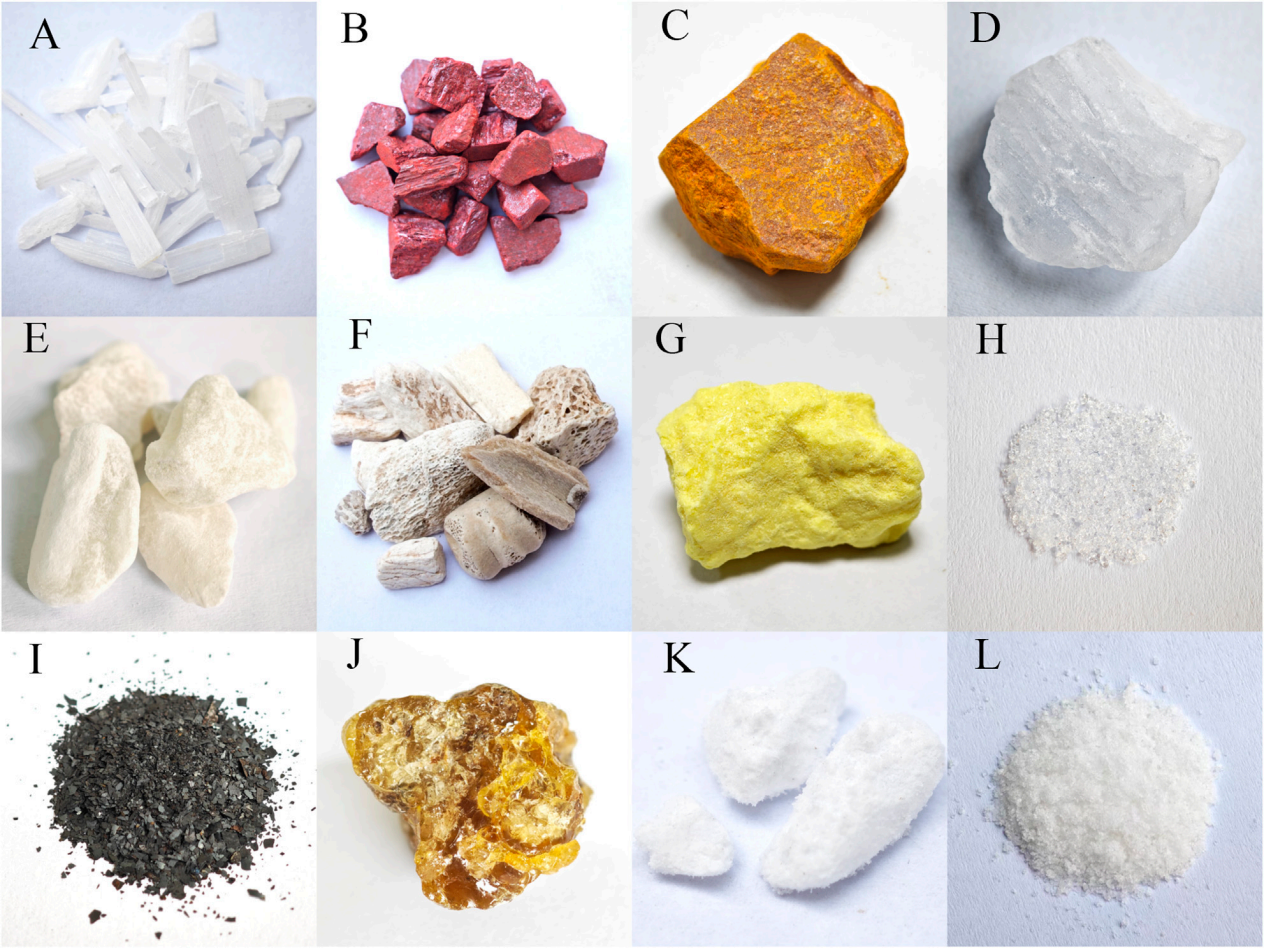
Representative specimens of the most frequently recorded historical mineral medicines in China and Japan (excluding salt). **(A)** Gypsum **(B)** Cinnabar **(C)** Realga **(D)** Alumen **(E)** Talcum **(F)** Dragon’s Bones **(G)** Sulfur **(H)** Calomel **(I)** Ferrum **(J)** Succinum **(K)** Mirabilite **(L)** Niter.

The most frequently recorded historical mineral medicines in China and Japan show a high degree of similarity. In Chinese historical medical texts, the top ten mineral medicines by number of occurrences are salt, gypsum, cinnabar, realgar, alumen, talcum, dragon’s bones, sulfur, calomel, and ferrum. In Japanese historical medical texts, the top ten are salt, gypsum, cinnabar, ferrum, talcum, succinum, mirabilite, dragon’s bones, realgar, and niter. Except for dragon’s bones, the other nine mineral medicines in Japan’s top ten have historical records of domestic production. Among the top ten in both countries, seven are identical—salt, gypsum, cinnabar, ferrum, talcum, dragon’s bones, and realgar. Based on the historical background of Kampo medicine and records of mineral production in Japan, the similarity in the top ten mineral medicines can be attributed, on the one hand, to the extensive adoption of mineral medicines from TCM into Kampo, and, on the other hand, to the availability of mineral resources in Japan, which ensured their practical use (Figure 7).

**FIGURE 8:**
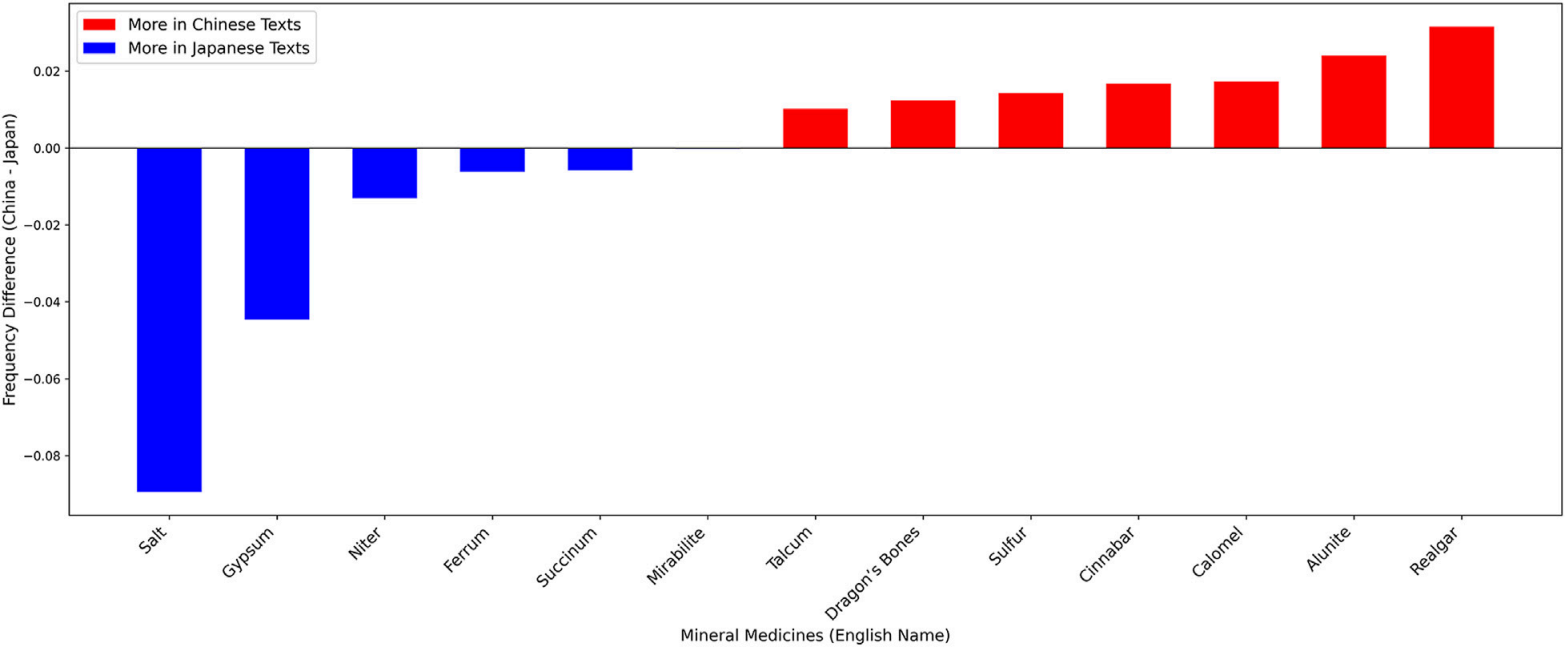
Top 10 mineral medicines: Frequency differences in Chinese and Japanese ancient medical texts.

To assess whether the occurrence frequencies of the most frequently recorded historical mineral medicines differ between China and Japan—particularly for the three items that appear in Japan’s top ten but not China’s (niter, succinum, and mirabilite)—we computed, using Python, the occurrence frequency of each top-ten mineral medicine in the historical texts of each country. We then constructed a differential index by subtracting the Japanese occurrence frequency from the Chinese occurrence frequency (China − Japan), which reflects the relative prominence of each mineral medicine across the two medical traditions. Positive values indicate greater prominence in China, whereas negative values indicate greater prominence in Japan. The results were visualized with Python and are presented in Figure 8.

The 13 non-overlapping commonly recorded mineral medicines in the medical history of China and Japan are salt, gypsum, cinnabar, ferrum, talcum, realgar, dragon’s bones, alumen, sulfur, calomel, niter, succinum, and mirabilite. Among them, six mineral medicines—salt, gypsum, niter, ferrum, succinum, and mirabilite—appear more frequently in Japanese historical medical texts, while the remaining seven occur more often in Chinese historical medical texts. Comparing the magnitude of frequency differences, salt and gypsum stand out, indicating that these two mineral medicines were used more frequently in Japanese medical history than in China. In addition, the three mineral medicines distinctive to Japan—niter, succinum, and mirabilite—also occur more frequently in Japanese historical medical texts, suggesting that their application in Kampo medicine may differ from that in TCM and embody local characteristics of Japan. Outside the set of commonly recorded mineral medicines, other examples, such as “hot spring,” also appear more frequently in Japanese historical medical texts, reflecting further distinctions between Kampo medicine and TCM.

### 3.2 Focused analysis of representative mineral medicines: mercury- and mercury-compound-based mineral medicines and gypsum

In the preceding cation-based classification analysis of mineral medicines, mercury-based mineral medicines ranked among the top three in terms of proportion of occurrences in historical medical texts of both China and Japan. As toxic mineral medicines, their high frequency of occurrence in the historical medical literature of both countries makes the pharmacological use of this category worthy of further investigation.

Referring to the ConPhyMP guidelines for grading plant extracts (Heinrich et al., 2022), gypsum was selected as another focal mineral medicine. It is included in the modern authoritative pharmacopoeias of both China and Japan and—excluding salt—was the highest-ranked common mineral medicine in historical records of both countries. The subsequent analysis focuses on the medicinal use of gypsum in historical Chinese and Japanese medical texts, as well as its continuity and transformation from ancient to modern times.

#### 3.2.1 Comparative analysis of the medicinal use of mercury- and mercury-compound-based mineral medicines

**FIGURE 9:**
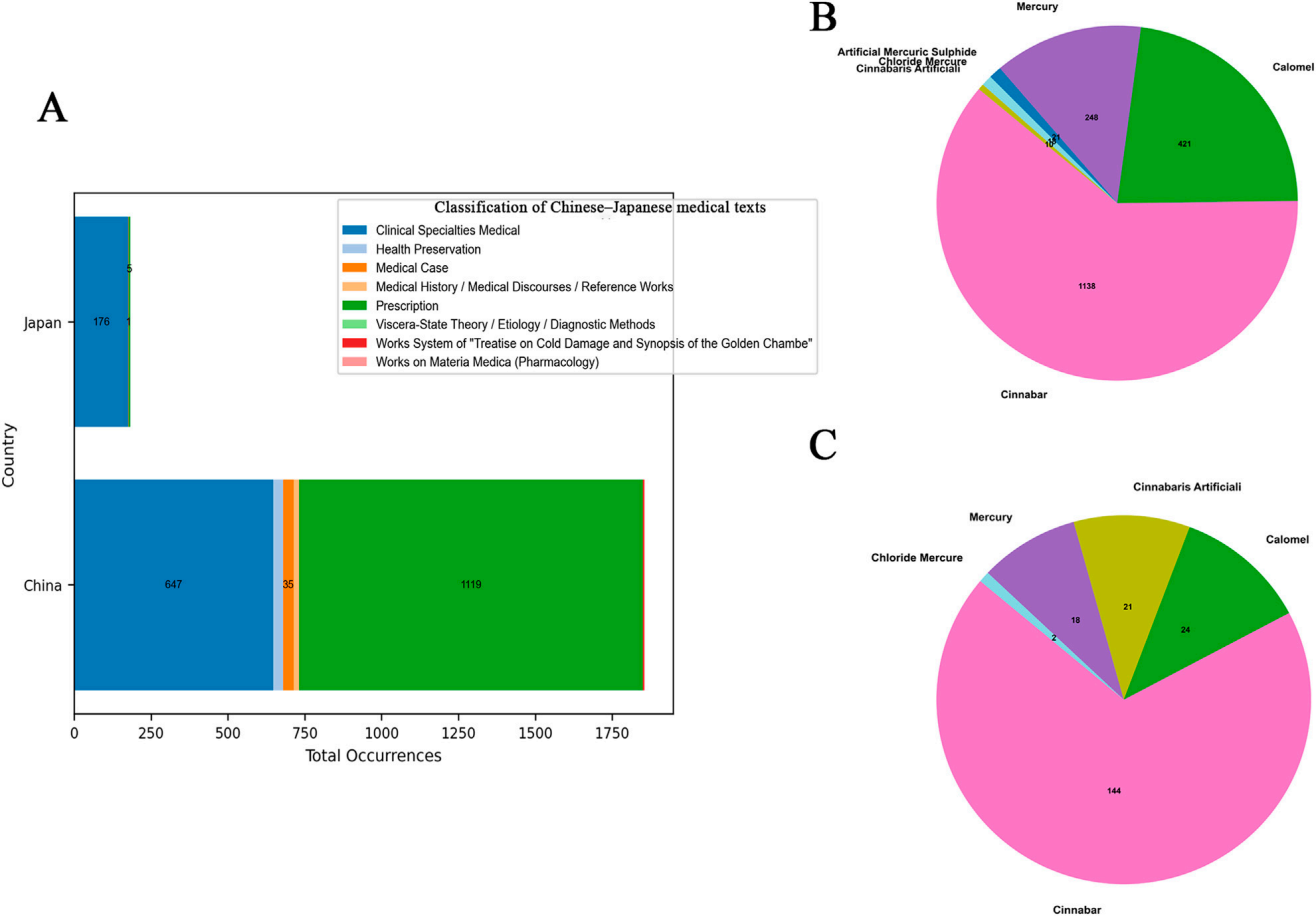
**(A)** Classification of Chinese -Japanese medical texts. **(B)** Composition of Mercury and Its Compounds-Based Mineral Medicines in China. **(C)** Composition of Mercury and Its Compounds-Based Mineral Medicines in Japan.

In China, medicinal information on mercury-based mineral medicines was obtained from 18 historical Chinese medical texts, most of which belonged to the “Prescription” and “Clinical Specialties Medical” categories. A total of 1,856 medicinal records were extracted, involving six mineral medicines: cinnabar, calomel, mercury, artificial mercuric sulphide, mercuric chloride, and artificial cinnabar (Figure 9).

In Japan, medicinal information on mercury-based mineral medicines was sourced from six historical Japanese medical texts, most of which belonged to the “Clinical Specialties Medical” category. A total of 209 medicinal records were extracted, involving five mineral medicines: cinnabar, calomel, artificial cinnabar, mercury, and mercuric chloride.

The types of mercury-based mineral medicines used in the history of traditional medicine in China and Japan were largely similar. In both Chinese and Japanese historical texts, cinnabar was the most frequently recorded mineral medicine in this category, indicating its central role among mercury-based mineral medicines.

The medical conditions treated with mercury-based mineral medicines in the historical records of both countries were also broadly similar. In historical Chinese texts, 189 conditions were documented as being treated with mercury-based mineral medicines or compound prescriptions containing them, while 98 such conditions were recorded in Japanese texts. Only two conditions—belching and excessive sexual indulgence—were documented exclusively in Japanese historical texts.

**FIGURE 10:**
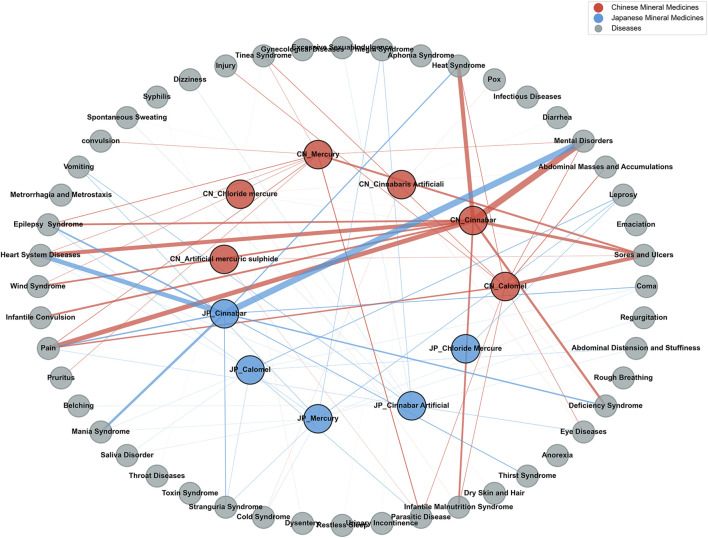
Network of strongly associated conditions for mercury-based mineral medicines in historical Chinese and Japanese medical texts.

For strong association analysis, the top ten conditions by number of occurrences for each of the eleven mercury- and mercury-compound-based mineral medicines recorded in the two countries were selected. A total of 35 strongly associated conditions were identified for China, and 25 for Japan, with 12 conditions shared between the two countries (Figure 10).

Statistical analysis was performed to identify the strongly associated conditions in China and Japan, along with their number of occurrences, the number of associated mineral medicine species, and the specific associated mineral medicines for all mercury-based mineral medicines (Table 3). Sores and ulcers and pain were the strongly associated conditions linked to all mercury-based mineral medicines in China, representing the core conditions treated with this category of medicines in historical Chinese medical texts. In Japan, no strongly associated conditions were linked to all mercury-based mineral medicines. The twelve strongly associated conditions shared by both countries can be regarded as conditions of general significance in the medical history of China and Japan, reflecting the common therapeutic applications of prescriptions containing mercury-based mineral medicines.

**TABLE 3:** Strongly associated conditions for mercury-based mineral medicines in historical Chinese and Japanese medical texts.

China				Japan			
Condition	Number of Occurrences	Number of Associated Mineral Medicines	Associated Mineral Medicines	Condition	Number of Occurrences	Number of Associated Mineral Medicines	Associated Mineral Medicines
Sores and Ulcers	345	6	Chloride mercure, Calomel, Mercury, Cinnabar, Artificial mercuric sulphide, Cinnabaris Artificiali	Epilepsy Syndrome	21	4	Chloride Mercure, Calomel, Mercury, Cinnabar
Pain	261	6	Chloride mercure, Calomel, Mercury, Cinnabar, Artificial mercuric sulphide, Cinnabaris Artificiali	Leprosy	17	4	Chloride Mercure, Calomel, Mercury, Cinnabar
Heart System Diseases	177	4	Calomel, Mercury, Cinnabar, Cinnabaris Artificiali	Abdominal Distension and Stuffiness	10	4	Cinnabar Artificial, Calomel, Mercury, Cinnabar
Infantile Malnutrition Syndrome	97	4	Chloride mercure, Calomel, Mercury, Cinnabar	Parasitic Disease	9	4	Cinnabar Artificial, Calomel, Mercury, Cinnabar
Parasitic Disease	96	4	Calomel, Mercury, Cinnabar, Artificial mercuric sulphide	Heart System Diseases	48	3	Cinnabar Artificial, Calomel, Cinnabar
Deficiency Syndrome	90	4	Calomel, Mercury, Cinnabar, Cinnabaris Artificiali	Mania Syndrome	27	3	Calomel, Mercury, Cinnabar
Emaciation	73	4	Chloride mercure, Calomel, Mercury, Cinnabar	Pain	18	3	Cinnabar Artificial, Calomel, Cinnabar
Toxin Syndrome	65	4	Calomel, Mercury, Cinnabar, Artificial mercuric sulphide	Deficiency Syndrome	17	3	Cinnabar Artificial, Mercury, Cinnabar
Eye Diseases	56	4	Calomel, Mercury, Cinnabar, Cinnabaris Artificiali	Vomiting	16	3	Cinnabar Artificial, Mercury, Cinnabar
Abdominal Masses and Accumulations	54	4	Chloride mercure, Calomel, Mercury, Cinnabar	Phlegm Syndrome	15	3	Cinnabar Artificial, Mercury, Cinnabar
Pruritus	43	4	Calomel, Mercury, Cinnabar, Artificial mercuric sulphide	Stranguria Syndrome	14	3	Calomel, Mercury, Cinnabar
Tinea Syndrome	35	4	Calomel, Mercury, Cinnabar, Artificial mercuric sulphide	Coma	12	3	Calomel, Mercury, Cinnabar
Abdominal Distension and Stuffiness	31	4	Chloride mercure, Calomel, Mercury, Cinnabar	Saliva Disorder	7	3	Calomel, Mercury, Cinnabar
Vomiting	25	4	Calomel, Mercury, Cinnabar, Cinnabaris Artificiali	Regurgitation	4	3	Cinnabar Artificial, Mercury, Cinnabar
Restless Sleep	20	4	Calomel, Mercury, Cinnabar, Cinnabaris Artificiali	Mental Disorders	63	2	Mercury, Cinnabar
Dysentery	18	4	Chloride mercure, Calomel, Mercury, Cinnabar	Thirst Syndrome	9	2	Cinnabar Artificial, Cinnabar
Anorexia	15	4	Chloride mercure, Calomel, Mercury, Cinnabar	Infectious Diseases	4	2	Calomel, Cinnabar
Syphilis	12	4	Calomel, Mercury, Cinnabar, Artificial mercuric sulphide	Spontaneous Sweating	4	2	Cinnabar Artificial, Cinnabar
Mental Disorders	277	3	Calomel, Mercury, Cinnabar	Dizziness	3	2	Cinnabar Artificial, Cinnabar
Heat Syndrome	192	3	Calomel, Mercury, Cinnabar	Heat Syndrome	11	1	Cinnabar
Epilepsy Syndrome	102	3	Calomel, Mercury, Cinnabar	Eye Diseases	4	1	Cinnabar Artificial
Infantile Convulsion	96	3	Calomel, Mercury, Cinnabar	Belching	2	1	Cinnabar Artificial
Wind Syndrome	86	3	Calomel, Mercury, Cinnabar	Cold Syndrome	2	1	Mercury
convulsion	47	3	Calomel, Mercury, Cinnabar	Excessive Sexual Indulgence	2	1	Cinnabar Artificial
Pox	31	3	Calomel, Cinnabar, Cinnabaris Artificiali	Aphonia Syndrome	1	1	Calomel
Cold Syndrome	30	3	Mercury, Cinnabar, Cinnabaris Artificiali				
Diarrhea	12	3	Calomel, Cinnabar, Cinnabaris Artificiali				
Throat Diseases	7	3	Calomel, Cinnabar, Artificial mercuric sulphide				
Spontaneous Sweating	5	3	Mercury, Cinnabar, Cinnabaris Artificiali				
Injury	23	2	Calomel, Cinnabar				
Dry Skin and Hair	3	2	Chloride mercure, Cinnabar				
Rough Breathing	2	2	Chloride mercure, Mercury				
Urinary Incontinence	2	1	Cinnabaris Artificiali				
Gynecological Diseases	1	1	Artificial mercuric sulphide				
Metrorrhagia and Metrostaxis	1	1	Artificial mercuric sulphide				

Note: The table is sorted in descending order by the number of associated mineral medicine species, and for cases with the same number of associated species, in descending order by the number of occurrences. The orange-shaded rows indicate the 12 strongly associated conditions shared by both China and Japan.

Considering both the number of occurrences and the number of associated mineral medicine species for the strongly associated conditions, six conditions were selected from the twelve shared strongly associated conditions between the two countries. These included pain, vomiting, heart system diseases, abdominal distension and stuffiness, parasitic disease, and deficiency syndrome. These represent the core conditions of general significance in the historical medical records of China and Japan that were treated with prescriptions containing mercury-based mineral medicines.

From historical Chinese medical texts, 1,799 prescriptions containing mercury-based mineral medicines were extracted, yielding a total of 677 co-occurring medicines. From historical Japanese medical texts, 169 such prescriptions were extracted, yielding a total of 257 co-occurring medicines. There were 240 co-occurring medicines shared between China and Japan, accounting for approximately 35.5% of the total co-occurring medicines in China and 93.4% in Japan.

Seventeen co-occurring medicines recorded in Japanese historical texts were not found in Chinese texts, reflecting the localized characteristics of Kampo medicine in Japan when applying mercury-based mineral medicines. These seventeen distinctive co-occurring medicines occurred infrequently and were mainly associated with cinnabar and mercury (Table 4).

**TABLE 4:** Co-occurring medicines unique to historical Japanese medical texts for mercury-based mineral medicines.

Co-occurring Medicines	Number of Occurrences	Number of Associated Mineral Medicines	Associated Mineral Medicines
Archemuschel	2	2	Mercury, Cinnabar
Astragali Radix Praeparata	1	1	Cinnabar
Barley	1	1	Cinnabaris Artificiali
Bean Blister Beetle	2	2	Mercury, Cinnabar
Colla Ichthyocolla	1	1	Cinnabar
Daturae Flos	1	1	Cinnabar
Dioscoreae Hypoglaucae Rhizoma	1	1	Cinnabar
Extract is made of juice of Kusnezoff Monkshood	2	2	Mercury, Cinnabar
Felis silvestris	2	2	Mercury, Cinnabar
Fragrant-flowered Garlic	1	1	Cinnabar
Gossypii Hirsuti Semen	1	1	Calomel
Isatidis Folium	1	1	Cinnabar
Margaritifera Concha	1	1	Cinnabar
Mume Nucleus	1	1	Calomel
Nelumbinis Plumula	2	2	Cinnabar
Pangolin Scales	2	2	Mercury, Cinnabar
Vespertilio	1	1	Cinnabar

**FIGURE 11:**
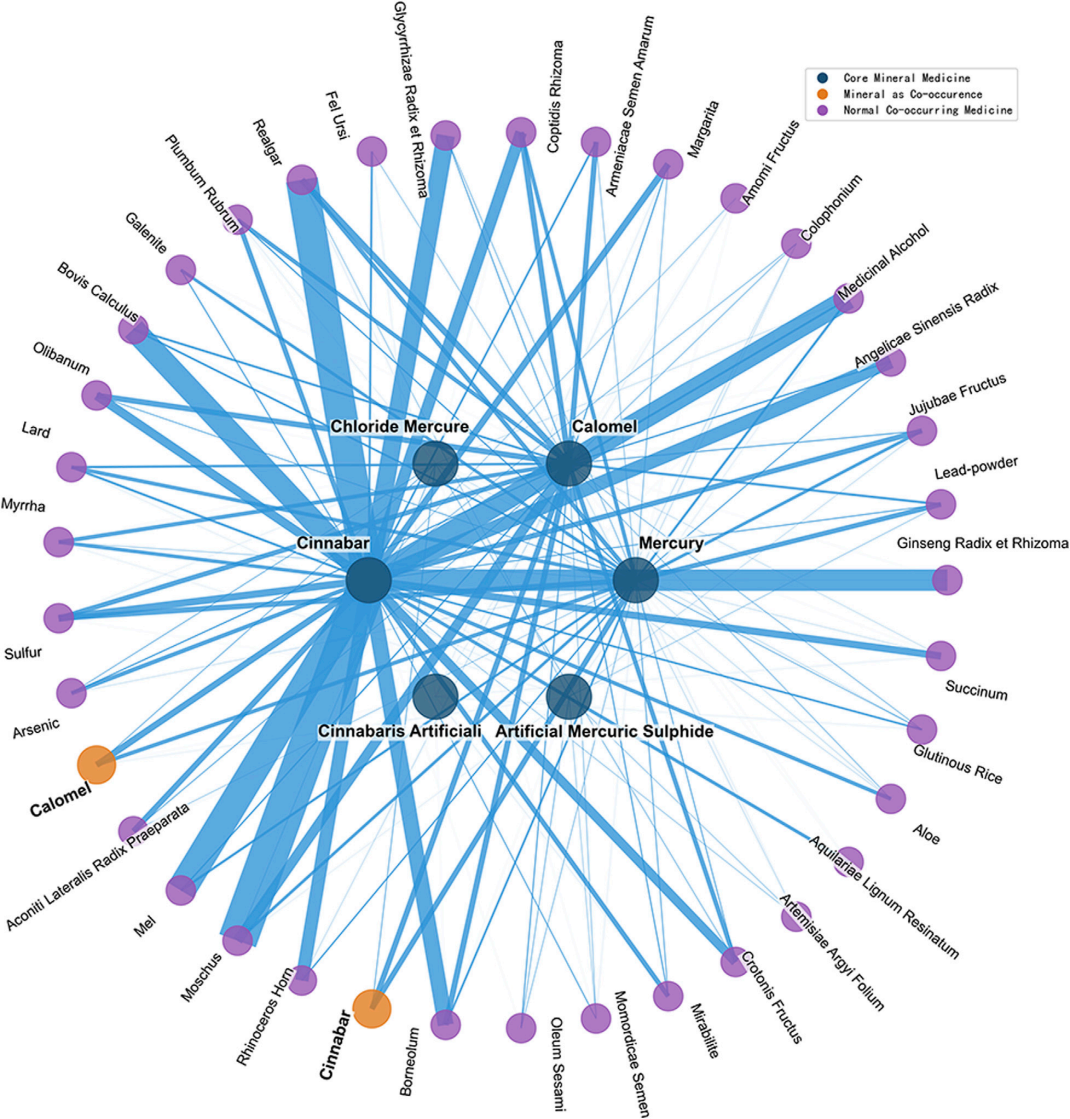
Network of strongly paired medicines for mercury-based mineral medicines in historical Chinese medical texts.

**FIGURE 12:**
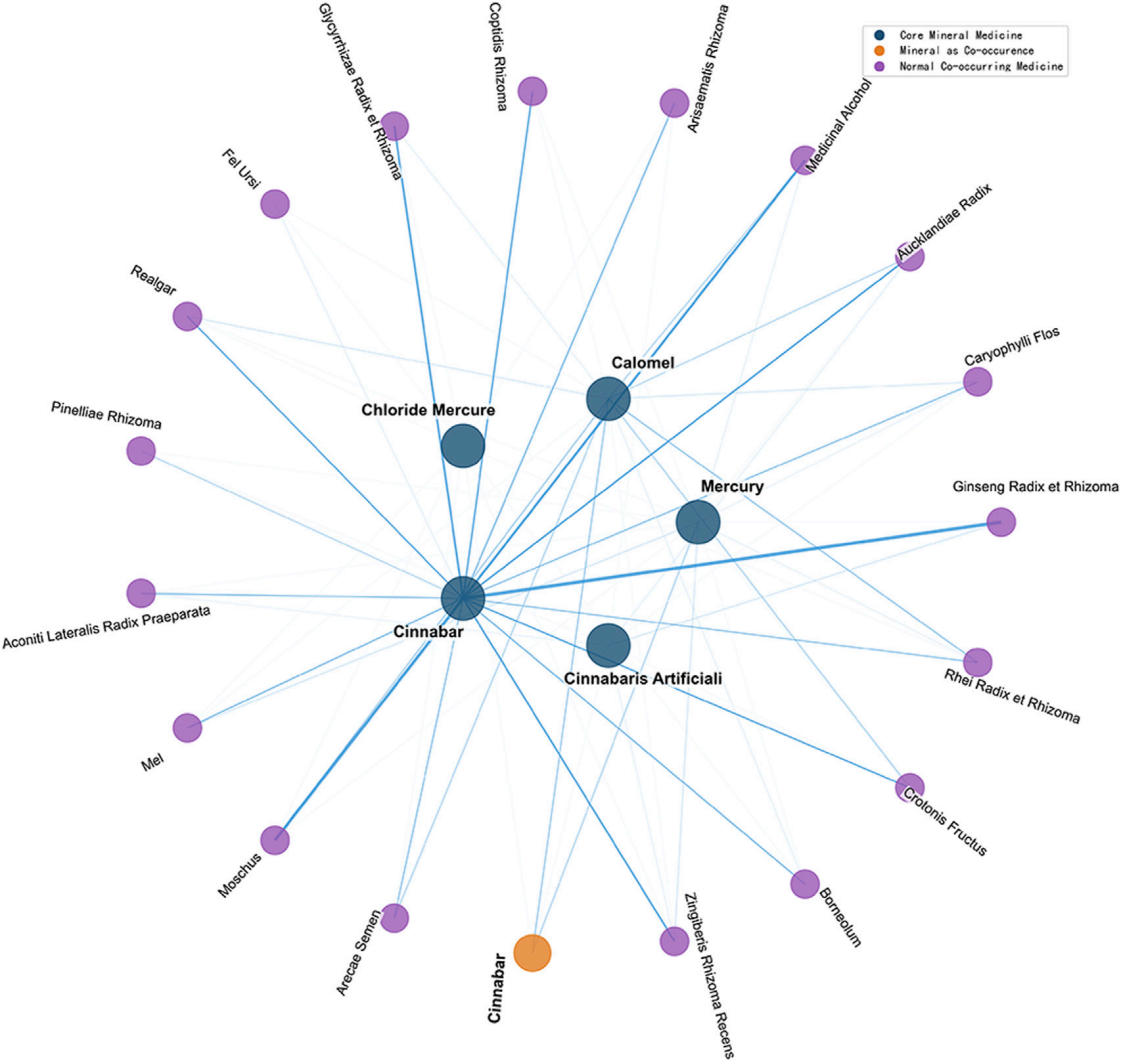
Network of strongly paired medicines for mercury-based mineral medicines in historical Japanese medical texts.

From the eleven mercury-based mineral medicines with documented medicinal information in historical Chinese and Japanese medical texts, the top ten co-occurring medicines by number of occurrences for each mineral medicine were selected. Among these, co-occurring medicines associated with three or more mineral medicine species were defined as strongly paired medicines. In total, 37 unique strongly paired medicines were identified in China, and 19 in Japan (Figures 11, 12).

Statistical analysis was conducted to determine the number of occurrences, the number of associated mineral medicine species, and the specific associated mineral medicines for all strongly paired medicines with mercury-based mineral medicines in China and Japan (Table 5). In historical Chinese medical texts, the only strongly paired medicine co-occurring with all six mercury-based mineral medicines was medicinal alcohol. In historical Japanese medical texts, the only strongly paired medicine co-occurring with all five mercury-based mineral medicines was Zingiber officinale Rosc. [Zingiberis Rhizoma Recens].

**TABLE 5:** Strongly paired medicines for mercury-based mineral medicines in historical Chinese and Japanese medical texts.

China				Japan			
Strongly Paired Medicines	Number of Occurrences	Number of Associated Mineral Medicines	Associated Mineral Medicines	Strongly Paired Medicines	Number of Occurrences	Number of Associated Mineral Medicines	Associated Mineral Medicines
Medicinal Alcohol	220	6	Chloride Mercure, Cinnabaris Artificiali, Calomel, Mercury, Artificial Mercuric Sulphide, Cinnabar	Zingiberis Rhizoma Recens	21	5	Chloride Mercure, Cinnabaris Artificiali, Calomel, Mercury, Cinnabar
Moschus	437	5	Chloride Mercure, Calomel, Mercury, Artificial Mercuric Sulphide, Cinnabar	Moschus	32	4	Chloride Mercure, Calomel, Mercury, Cinnabar
Realgar	360	5	Chloride Mercure, Calomel, Mercury, Artificial Mercuric Sulphide, Cinnabar	Aucklandiae Radix	21	4	Cinnabaris Artificiali, Calomel, Mercury, Cinnabar
Borneolum	198	5	Cinnabaris Artificiali, Calomel, Mercury, Artificial Mercuric Sulphide, Cinnabar	Crotonis Fructus	18	4	Cinnabaris Artificiali, Calomel, Mercury, Cinnabar
Sulfur	150	5	Chloride Mercure, Calomel, Mercury, Artificial Mercuric Sulphide, Cinnabar	Rhei Radix et Rhizoma	16	4	Chloride Mercure, Calomel, Mercury, Cinnabar
Olibanum	132	5	Chloride Mercure, Calomel, Mercury, Artificial Mercuric Sulphide, Cinnabar	Realgar	16	4	Chloride Mercure, Calomel, Mercury, Cinnabar
Calomel	96	5	Chloride Mercure, Cinnabaris Artificiali, Mercury, Artificial Mercuric Sulphide, Cinnabar	Caryophylli Flos	13	4	Cinnabaris Artificiali, Calomel, Mercury, Cinnabar
Plumbum Rubrum	89	5	Chloride Mercure, Calomel, Mercury, Artificial Mercuric Sulphide, Cinnabar	Mel	13	4	Chloride Mercure, Calomel, Mercury, Cinnabar
Jujubae Fructus	83	5	Chloride Mercure, Calomel, Mercury, Artificial Mercuric Sulphide, Cinnabar	Cinnabar	12	4	Chloride Mercure, Cinnabaris Artificiali, Calomel, Mercury
Lard	77	5	Chloride Mercure, Calomel, Mercury, Artificial Mercuric Sulphide, Cinnabar	Borneolum	12	4	Chloride Mercure, Calomel, Mercury, Cinnabar
Myrrha	71	5	Chloride Mercure, Calomel, Mercury, Artificial Mercuric Sulphide, Cinnabar	Ginseng Radix et Rhizoma	33	3	Cinnabaris Artificiali, Mercury, Cinnabar
Succinum	69	5	Chloride Mercure, Cinnabaris Artificiali, Calomel, Mercury, Cinnabar	Medicinal Alcohol	26	3	Calomel, Mercury, Cinnabar
Glutinous Rice	33	5	Chloride Mercure, Cinnabaris Artificiali, Calomel, Mercury, Cinnabar	Glycyrrhizae Radix et Rhizoma	20	3	Chloride Mercure, Calomel, Cinnabar
Momordicae Semen	20	5	Chloride Mercure, Calomel, Mercury, Artificial Mercuric Sulphide, Cinnabar	Coptidis Rhizoma	15	3	Calomel, Mercury, Cinnabar
Bovis Calculus	227	4	Chloride Mercure, Calomel, Mercury, Cinnabar	Arecae Semen	12	3	Chloride Mercure, Calomel, Cinnabar
Coptidis Rhizoma	179	4	Chloride Mercure, Calomel, Mercury, Cinnabar	Aconiti Lateralis Radix Praeparata	11	3	Cinnabaris Artificiali, Mercury, Cinnabar
Angelicae Sinensis Radix	138	4	Chloride Mercure, Calomel, Mercury, Cinnabar	Arisaematis Rhizoma	10	3	Chloride Mercure, Cinnabaris Artificiali, Cinnabar
Crotonis Fructus	128	4	Chloride Mercure, Calomel, Mercury, Cinnabar	Pinelliae Rhizoma	6	3	Cinnabaris Artificiali, Mercury, Cinnabar
Cinnabar	99	4	Chloride Mercure, Calomel, Mercury, Artificial Mercuric Sulphide	Fel Ursi	4	3	Chloride Mercure, Calomel, Cinnabar
Lead-powder	75	4	Calomel, Mercury, Artificial Mercuric Sulphide, Cinnabar				
Margarita	73	4	Cinnabaris Artificiali, Calomel, Mercury, Cinnabar				
Mirabilite	60	4	Cinnabaris Artificiali, Calomel, Mercury, Cinnabar				
Aconiti Lateralis Radix Praeparata	60	4	Cinnabaris Artificiali, Calomel, Mercury, Cinnabar				
Arsenic	55	4	Chloride Mercure, Calomel, Mercury, Cinnabar				
Aloe	43	4	Chloride Mercure, Calomel, Mercury, Cinnabar				
Aquilariae Lignum Resinatum	32	4	Calomel, Mercury, Artificial Mercuric Sulphide, Cinnabar				
Oleum Sesami	22	4	Calomel, Mercury, Artificial Mercuric Sulphide, Cinnabar				
Colophonium	16	4	Calomel, Mercury, Artificial Mercuric Sulphide, Cinnabar				
Artemisiae Argyi Folium	13	4	Calomel, Mercury, Artificial Mercuric Sulphide, Cinnabar				
Amomi Fructus	8	4	Cinnabaris Artificiali, Calomel, Mercury, Cinnabar				
Mel	274	3	Calomel, Mercury, Cinnabar				
Ginseng Radix et Rhizoma	181	3	Calomel, Mercury, Cinnabar				
Glycyrrhizae Radix et Rhizoma	165	3	Calomel, Mercury, Cinnabar				
Rhinoceros Horn	125	3	Calomel, Mercury, Cinnabar				
Armeniacae Semen Amarum	66	3	Calomel, Mercury, Cinnabar				
Galenite	36	3	Calomel, Mercury, Cinnabar				
Fel Ursi	24	3	Cinnabaris Artificiali, Calomel, Cinnabar				

Note: The table is sorted in descending order by the number of associated mineral medicine species, and for cases with the same number of associated species, in descending order by the number of occurrences. The orange-shaded rows indicate the 12 strongly paired medicines shared by both China and Japan.

Among China’s strongly paired medicines, nine were mineral medicines: realgar, sulfur, calomel, succinum, cinnabar, lead-powder, mirabilite, arsenic, and galenite. Among Japan’s strongly paired medicines, two were mineral medicines: realgar and cinnabar. Mercury-based mineral medicines in China and Japan were also paired with each other, with cinnabar being the most frequently co-paired mineral medicine.

Twelve strongly paired medicines for mercury-based mineral medicines were shared between China and Japan: medicinal alcohol, Moschus berezovskii Flerov [Moschus], realgar, Cinnamomum camphora (L.) Presl [Borneolum], Coptis chinensis Franch. [Coptidis Rhizoma], Croton tiglium L. [Crotonis Fructus], cinnabar, Aconitum carmichaelii Debx. [Aconiti Lateralis Radix Praeparata], Apis cerana Fabricius [Mel], Panax ginseng C.A. Mey. [Ginseng Radix et Rhizoma], Glycyrrhiza uralensis Fisch. [Glycyrrhizae Radix et Rhizoma], and Ursus arctos [Fel Ursi]. These twelve strongly paired medicines can be regarded as the core medicines of general significance in the historical medical records of China and Japan associated with prescriptions containing mercury-based mineral medicines.

Processing (paozhi) is one of the key methods for reducing the toxicity of mineral medicines. The processing methods for mercury-based mineral medicines recorded in historical Chinese medical texts were more diverse than those in Japanese texts. In both countries, the most frequently recorded processing method was finely ground, followed by levigation. In Chinese historical texts, nine processing methods were documented: boiled with Ephedra water, decocted with lead, finely ground, levigation, long decoction until charred, oil-frying, processed with Semiaquilegia adoxoides (DC.) Makino [Herba Semiaquilegiae], processing with Herba Semiaquilegiae, and roasting method. Among these, finely ground and levigation were the most common, accounting for 77.6% and 11.7% of all medicinal records, respectively. In Japanese historical texts, only two processing methods were recorded: finely ground and levigation, accounting for 44% and 11% of all medicinal records, respectively.

For the administration of prescriptions containing mercury-based mineral medicines, oral administration was the predominant route in both countries. In Chinese historical texts, nine routes of administration were documented: buccal administration, ear packing, external use, eye instillation, fumigation, incineration, oral administration, tooth wiping, and wearing. Among these, oral administration and external use were the most frequent, accounting for 66.2% and 27.2% of all medicinal records, respectively. In Japanese historical texts, only two routes of administration were recorded: oral administration and external use, accounting for 91.4% and 2.4% of all medicinal records, respectively.

Interestingly, despite their toxicity, mercury-based mineral medicines were explicitly and repeatedly documented in both countries as being administered to pediatric patients. In historical Chinese medical texts, 427 references to “Xiaoer (children)” or similar expressions were identified among 1,856 medicinal records, involving four mercury-based mineral medicines: mercuric chloride, calomel, mercury, and cinnabar. In historical Japanese medical texts, three such references were found among 209 medicinal records, involving calomel, mercury, and cinnabar. Although the overall proportion was low, the explicit indication of pediatric use is noteworthy.

In addition, historical medical texts in both countries frequently recorded the use of cinnabar in the form of “Weiyi (clothing)” (coating other substances) after being finely ground. In Chinese historical texts, this practice was recorded 93 times among 1,138 medicinal records for cinnabar. In Japanese historical texts, 26 such instances were found among 114 medicinal records for cinnabar. This practice may be related to the red color of cinnabar, which, according to traditional medical theory, guides the medicine to the heart meridian.

#### 3.2.2 Comparative analysis of the medicinal use of gypsum

In China, medicinal information on gypsum was obtained from 25 historical Chinese medical texts, most of which belonged to the “Prescription” and “Clinical Specialties Medical” categories. A total of 1,297 medicinal records related to gypsum were extracted, of which 984 prescriptions—including those using gypsum alone—contained gypsum.

**FIGURE 13:**
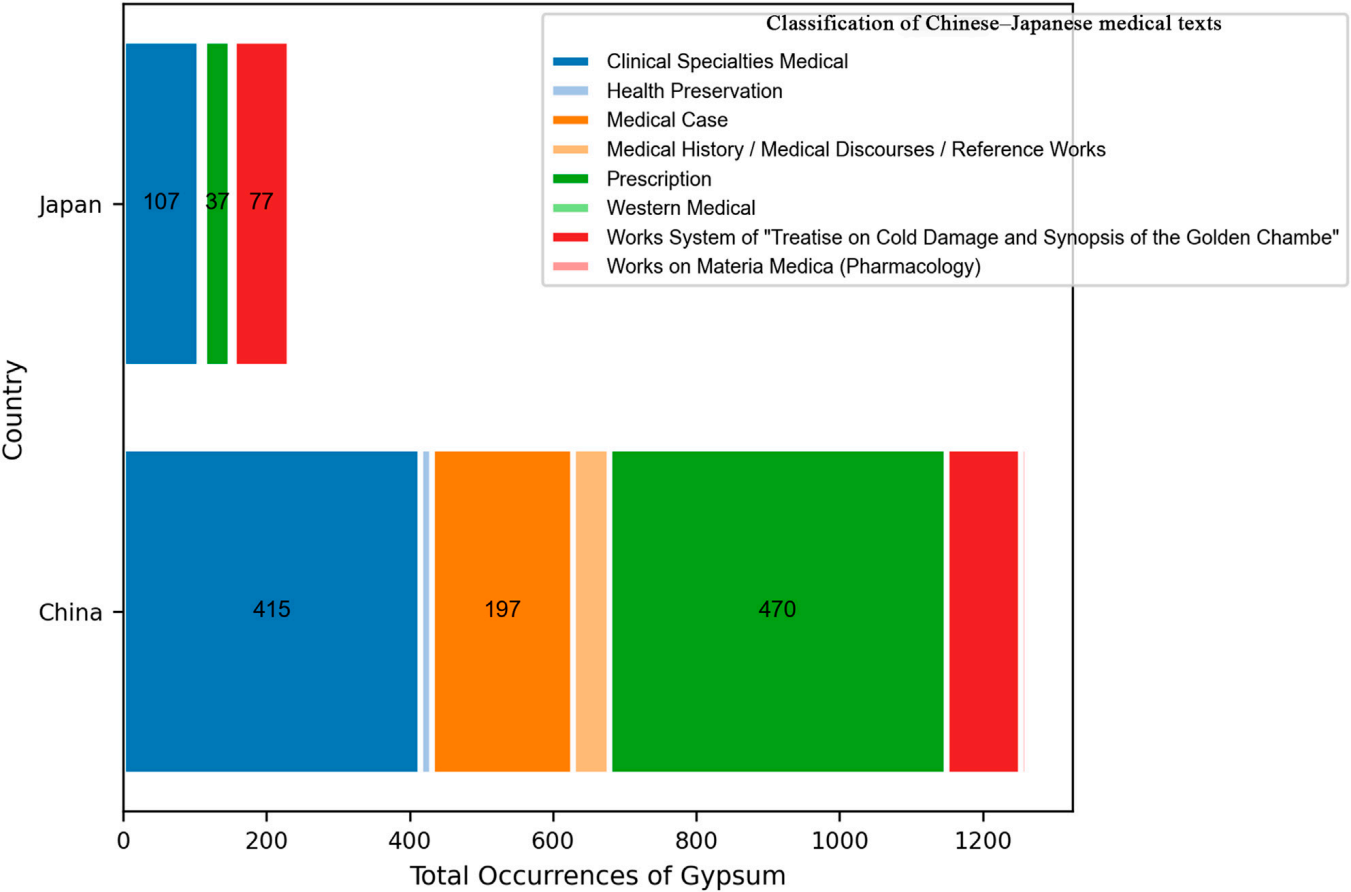
Classification of Chinese -Japanese medical texts.

In Japan, medicinal information on gypsum was sourced from nine historical Japanese medical texts, most of which belonged to the “Clinical Specialties Medical” and “Works System of Treatise on Cold Damage and Synopsis of the Golden Chamber” categories. A total of 240 medicinal records related to gypsum were extracted, of which 130 prescriptions—including those using gypsum alone—contained gypsum (Figure 13).

In historical Chinese medical texts, a total of 429 co-occurring medicines were recorded with gypsum, while in historical Japanese medical texts, 168 such medicines were documented. Among the co-occurring medicines with gypsum in both countries, 140 were shared, accounting for 83.3% of the total co-occurring medicines in Japan and 32.6% in China.

Japan had 25 unique co-occurring medicines with gypsum, all of which had a number of occurrences in the single digits. Among these, ten were animal medicines (e.g., aranea ventricosa, egg, hirudo) and three were mineral medicines (calomel, mercury, Natrii Sulfas Exsiccatus), reflecting localized characteristics of gypsum pairings in Japanese Kampo medicine.

**FIGURE 14:**
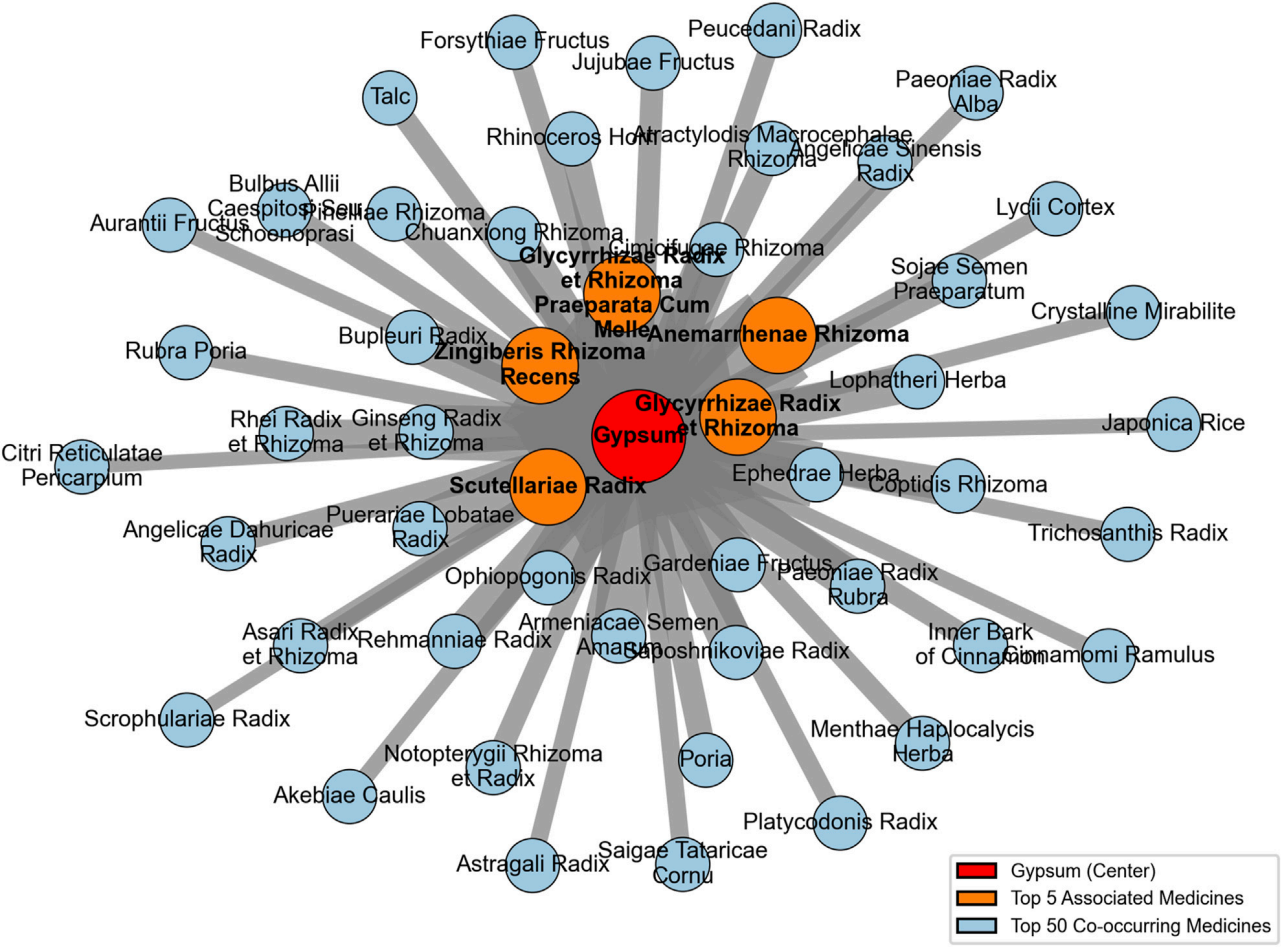
Network of the top 50 co-occurring medicines with gypsum in historical Chinese medical texts.

Among the 429 co-occurring medicines with gypsum recorded in historical Chinese medical texts, the top five by strength of association (in descending order) were: Glycyrrhizae Radix et Rhizoma, Scutellaria baicalensis Georgi [Scutellariae Radix], Zingiberis Rhizoma Recens, Anemarrhena asphodeloides Bge. [Anemarrhenae Rhizoma], and Glycyrrhiza uralensis Fisch. [Glycyrrhizae Radix et Rhizoma Praeparata Cum Melle] (see Figure 14).

**FIGURE 15:**
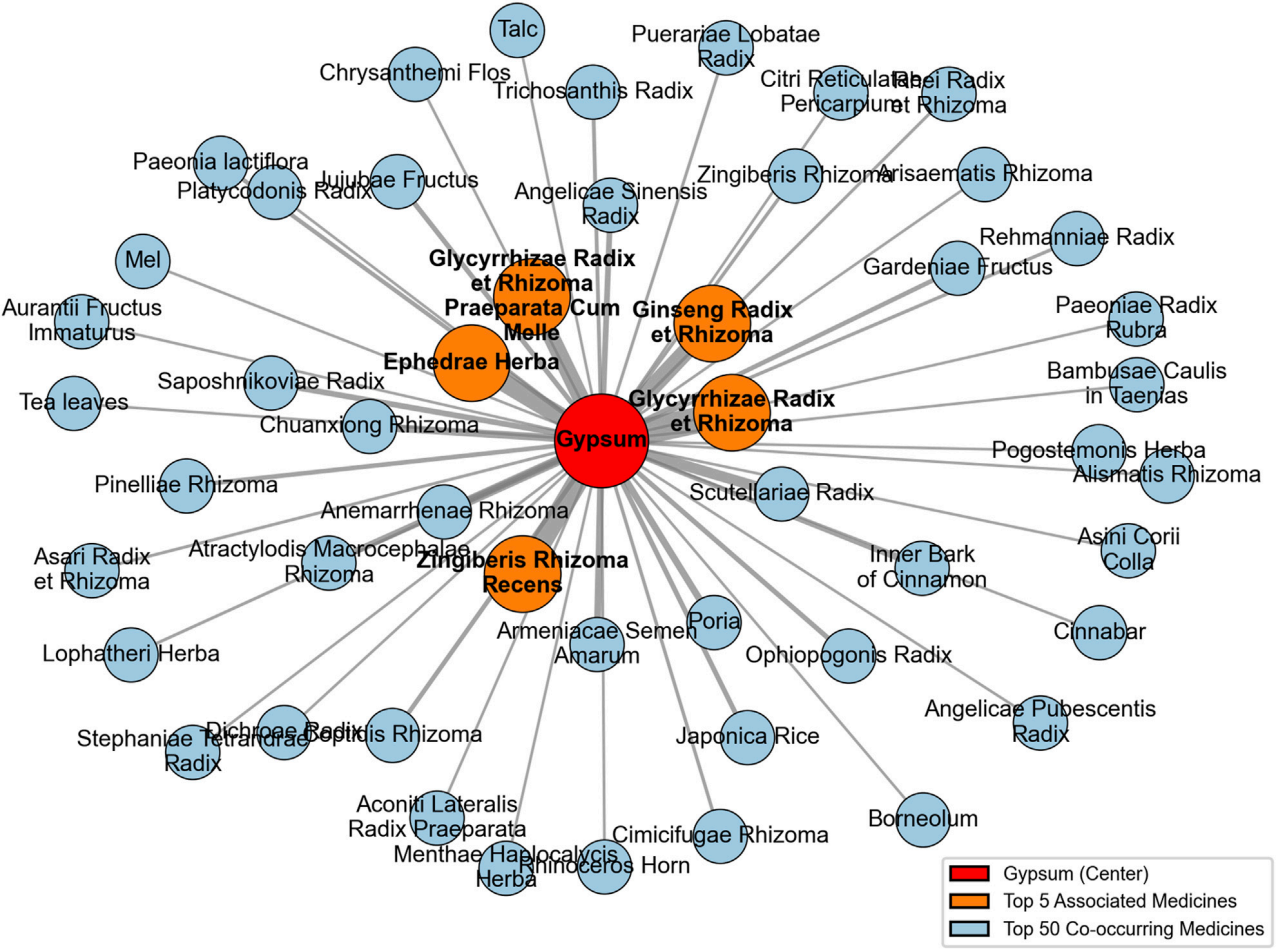
Network of the top 50 co-occurring medicines with gypsum in historical Japanese medical texts.

Among the 168 co-occurring medicines with gypsum recorded in historical Japanese medical texts, the top five by strength of association (in descending order) were: Glycyrrhizae Radix et Rhizoma, Zingiberis Rhizoma Recens, Ephedra sinica Stapf [Ephedrae Herba], Ginseng Radix et Rhizoma, and Glycyrrhizae Radix et Rhizoma Praeparata Cum Melle (see Figure 15).

Three of the top five strongly associated medicines were the same in both countries, while two differed. In Japanese historical texts, Scutellariae Radix and Anemarrhenae Rhizoma ranked sixth and seventh, respectively, for co-occurrence with gypsum, whereas in Chinese historical texts, Ephedrae Herba and Ginseng Radix et Rhizoma ranked sixth and eighth, respectively. Overall, the differences between the strongly associated medicines with gypsum in China and Japan were minimal.

Compared with Japan, Chinese historical medical texts recorded a greater diversity of medicines paired with gypsum. The combinations involving gypsum in Japanese historical medical texts were heavily influenced by Chinese medicine. Although there were some unique features, the strongly paired medicines overlapped substantially with those in China, reflecting that the pairing patterns of gypsum in Japanese Kampo medicine were largely inherited from TCM.

**FIGURE 16:**
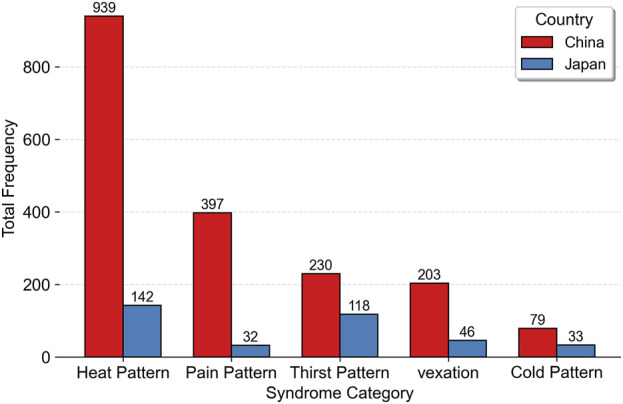
Top five primary conditions treated with gypsum and gypsum-containing prescriptions in historical Chinese and Japanese medical texts.

Using a word frequency analysis approach, the disease descriptions recorded in historical Chinese and Japanese medical texts were segmented, manually cleaned, and classified. The main conditions treated with gypsum or gypsum-containing prescriptions in the two countries were then identified. From these, the top five conditions by frequency of occurrence were selected for a comparative analysis of the primary therapeutic indications of gypsum as documented in the historical medical texts of China and Japan (see Figure 16).

In both Chinese and Japanese records, the most frequently mentioned conditions pattern treated with gypsum was “heat pattern” (re zheng). This category includes both internally generated heat, such as “body heat” (shen re), and externally induced heat, such as “summer heat” (shu re). The intensity of heat symptoms varied across descriptions, ranging from severe expressions like re jue (“heat syncope”) and zhuang re (“intense fever”) to milder forms such as yu re (“residual heat”). The affected areas were most often located in the upper burner—including the mouth, nose, throat, heart, and lungs—and in the middle burner (especially the stomach and yangming channel). In some cases, the location of the fever was vaguely described as generalized “body heat.”

In the Chinese texts, the second most frequently treated condition with gypsum was pain, with headache being the most common, followed by references to body pain, toothache, and lower back pain. In contrast, pain ranked last among the top five indications in the Japanese texts, revealing a notable difference between the two traditions—though headache remained the predominant form of pain described in both. This suggests that while the descriptions of pain-related indications for gypsum were similar in historical Chinese and Japanese medical texts, pain was given greater clinical emphasis in China than in Japan.

In both Chinese and Japanese historical medical texts, thirst pattern (ke zheng) frequently appeared alongside heat pattern (re zheng) and vexation (fan zheng). This co-occurrence not only reveals the underlying pathogenic mechanism of thirst, but also reflects the severity of both thirst and heat patterns. The descriptions related to vexation further indicate that historical texts from both countries paid attention to concurrent mental or emotional symptoms when documenting disease presentations.

It is noteworthy that cold pattern also appeared among the top five conditions associated with gypsum in both Chinese and Japanese historical medical texts. At first glance, this seems to contradict gypsum’s intrinsic cold nature and its role in clearing heat. However, the relevant descriptions in both corpora often refer to mixed cold-heat syndromes, such as exterior heat with interior cold (biao re li han), alternating cold and heat (wang lai han re), and fever with aversion to cold (fa re e han). The prominence of cold pattern as one of gypsum’s main indications suggests that gypsum was frequently paired with warming botanical drugs, such as Zingiberis Rhizoma Recens, which, as noted earlier, was among the top co-occurring substances in both corpora. This pairing indicates its use in treating conditions involving simultaneous cold and heat symptoms.

In the modern Pharmacopoeia of the People’s Republic of China, gypsum is described as having the functions of clearing heat, draining fire, relieving vexation, and quenching thirst. It is indicated for febrile diseases due to exogenous pathogens, high fever with vexation and thirst, lung heat with wheezing and cough, stomach fire excess, headache, and toothache (National Medical Products Administration of China, 2025). These indications align closely with the primary disease patterns identified in this study based on historical records. However, the use of gypsum for various types of pain, as documented in historical Chinese medical texts, appears to be more diverse than in the modern pharmacopoeia. In addition, the pharmacopoeia does not mention the traditional use of gypsum in combination with other substances to treat mixed cold-heat patterns. Overall, while gypsum remains a formally recognized mineral medicine in modern Chinese medical practice, its current indications have been slightly narrowed in comparison to its broader historical applications.

In the modern Japanese Pharmacopoeia, gypsum and exsiccated gypsum are described in terms of physical characteristics, identification methods, purity standards, and storage requirements, but no therapeutic functions or indications are provided (Ministry of Health and Labour and Welfare of Japan, 2021). Contemporary Kampo medicine literature in Japan states that gypsum has heat-clearing effects and helps to maintain body fluids. It is commonly used to treat symptoms such as persistent high fever, hyperthermia, dehydration, and intense thirst caused by exogenous heat or heatstroke. It is frequently combined with Anemarrhenae Rhizoma in the classical formula Baihu-tang (White Tiger Decoction) (Tanaka, 2020).

Compared with the usage characteristics identified in this study from historical Japanese medical texts, modern Kampo medicine continues to recognize gypsum’s efficacy in treating heat and thirst patterns, but largely overlooks its historical applications in treating pain, vexation, and cold patterns. Additionally, while the combination of gypsum with zhi mu is mentioned, this practice is generally based on clinical experience rather than supported by quantitative analysis or statistical validation.

Within the scope of this study, the conditions treated with gypsum and its combination formulas, as recorded in historical Chinese and Japanese medical texts, show no substantial differences between the two countries. While both the modern authoritative pharmacopoeias of China and Japan have retained gypsum as a commonly used medicinal substance, the range of its therapeutic indications has been reduced in both contexts—more notably in Japan than in China.

## 4 Discussion

### 4.1 Historical exploration

Unlike previous historical studies that focused on a single country, a limited number of historical texts, or a few mineral medicines, this study—building on the intrinsic connections between TCM and Japanese Kampo medicine—selected representative medical texts from different historical periods in both China and Japan to conduct a cross-era and cross-national investigation of mineral medicines. The dataset, derived from 56 historical medical texts, covers approximately 1,644 years of history and is both broad in scope and representative.

The selected texts encompass most major categories of traditional medical literature, with mineral medicine data most frequently sourced from “Prescription”-type works in both countries. The selection emphasized historical representativeness and diversity of text types, rather than matching word counts between Chinese and Japanese sources—resulting in notable textual length differences. Therefore, the comparative analysis of mineral medicines between the two countries is conducted relative to each country’s internal distribution.

A total of 109 mineral medicines were recorded in the Chinese corpus, and 100 in the Japanese corpus, with 97 species in common—indicating that Kampo medicine has extensively adopted the mineral materia medica of TCM. Based on cation classification, the Chinese corpus contains 16 categories and the Japanese corpus 14. The top three categories by occurrence are identical in both countries, although their order differs slightly: calcium compounds rank first in China, sodium compounds in Japan—a difference that may be related to Japan’s distinct natural environment. Notably, mercury- and mercury-compound-based mineral medicines rank third in both countries, their high toxicity and frequent usage forming a striking historical feature.

Using occurrence counts, the ten most frequently recorded mineral medicines in each country were identified as the most common historical mineral medicines. Seventy percent of these overlap between China and Japan, reflecting both the influence of TCM on Kampo medicine and the role of Japanese natural resources in shaping local mineral medicine applications. In Japan, salt and gypsum appear more frequently than in China, while niter, succinum, and mirabilite—unique to Japan’s top-ten list—also occur at higher rates. This suggests that Japan, while inheriting much from TCM, adapted mineral medicine use to local conditions. The specific reasons for these higher frequencies in Japan, however, lie beyond the scope of this study and warrant future research.

The study selected mercury- and mercury-compound-based mineral medicines—representing both high toxicity and high frequency—for a meso-level analysis, focusing on the disease conditions treated by their formulas and their co-occurring medicines. China recorded six such medicines, Japan five. Considering the top ten conditions and top ten co-occurring medicines for each mineral medicine, as well as the number of medicines associated with each condition or co-occurring medicine, six core conditions and twelve core strongly paired medicines common to both countries were identified. Characteristics of processing methods and administration routes were also examined.

At the micro level, gypsum—second only to salt in occurrence in both countries and recorded in both modern pharmacopoeias—was analyzed for its medicinal characteristics. The analysis covered the main conditions treated by gypsum and gypsum-containing formulas, as well as co-occurring medicines. Since modern pharmacopoeias do not record co-occurring medicines for gypsum, only historical–modern comparisons of main conditions were made. These comparisons show that the scope of gypsum’s indications in modern pharmacopoeias is narrower than that recorded in historical texts.

### 4.2 Modern implications

This study, through large-scale data analysis, has outlined the overall landscape of mineral medicines in historical Chinese and Japanese medical texts, as well as the characteristic therapeutic indications and co-occurring medicines of mercury- and mercury-compound-based mineral medicines and gypsum. The results provide a strong data-driven reference for future research on mineral medicines.

The 2025 edition of the Pharmacopoeia of the People’s Republic of China includes 23 mineral medicines, among which 7—cinnabar, gypsum, realgar, alumen, talcum, sulfur, and calomel—are identified in this study as among the most frequently recorded mineral medicines in Chinese medical history. In contrast, the 2021 edition of the Japanese Pharmacopoeia records only three mineral medicines: gypsum, its derivative exsiccated gypsum, and talcum.

Compared with the more than one hundred mineral medicines documented in historical Chinese and Japanese medical texts, the modern pharmacopoeias of both countries inherit only a small portion of the ancient mineral materia medica. This indicates that abundant mineral medicine resources remain to be further explored. The high-frequency mineral medicines identified in this study—particularly those historically common in both countries—are worthy candidates for further pharmacological investigation.

Our analysis of mercury- and mercury-compound-based mineral medicines shows that medicines within the same category indeed share commonalities in both therapeutic indications and strongly paired medicines. More than a dozen other types of mineral medicines recorded in historical Chinese and Japanese medical texts remain underexplored. Furthermore, both Chinese and Japanese historical texts repeatedly record the use of mercury- and mercury-compound-based mineral medicines in treating pediatric conditions—an aspect whose significance and feasibility merit further research.

Previous studies note, for instance, that in China’s Qin and Han periods, prescriptions used a mixture of mercury and realgar to treat scabies (Zhao et al., 2022). In our study, scabies falls under the category of Parasitic Disease, and Parasitic Disease together with realgar are identified as one of the core strong-associated conditions and core strongly paired medicines for mercury- and mercury-compound-based mineral medicines in both countries, which aligns closely with our findings.

This study has focused on therapeutic indications and co-occurring medicines for mercury- and mercury-compound-based mineral medicines and gypsum, treating each dimension independently without conducting integrated association analysis. Conducting experimental research to investigate the associations between the strongly associated conditions and strongly paired medicines identified here would be a promising future direction.

Existing research on co-occurring medicines notes, for example, that Zingiberis Rhizoma Recens, Ginseng Radix et Rhizoma, and Glycyrrhizae Radix et Rhizoma can mitigate the toxicity of Aconitum carmichaelii Debx. [Aconiti Radix] (Xiang et al., 2021). In the context of mineral medicines, baicalin has been reported to reduce the toxicity of cinnabar (Su et al., 2017), and gypsum is frequently combined with Anemarrhena asphodeloides (Tanaka, 2020). These studies show that research on co-occurring medicines often focuses on strategies to reduce toxicity or enhance efficacy, but rarely addresses the criteria for selecting co-occurring medicines.

By contrast, the co-occurring medicines identified in this study are derived from large-scale data analysis rather than subjective expert opinion, thereby providing a robust, data-backed foundation for future research aimed at toxicity mitigation, efficacy enhancement, and other related directions.

## Data Availability

The original contributions presented in the study are included in the article/Supplementary Material, further inquiries can be directed to the corresponding author.

## Footnotes

1 URL: www.xn--cesp9b.net/

2 The mineral medicines in parentheses are the two most frequently recorded representatives within each compound category

## References

[B1] Bau D. H. Nowak M. Lu D. J. Wang Q. C. Fitzgerald M. Zhang H. (2025). The outcast of medicine: metals in medicine--from traditional mineral medicine to metallodrugs. Front. Pharmacol. (07), 01–23. 10.3389/fphar.2025.1542560 PMC12010122 40260378

[B2] Brekhman I. I. Grinevitch M. A. (1981). Oriental medicine: a computerized study of complex recipes and their components: analysis of prescriptions used in the traditional medicine of Japan and Korea. Am. J. Chin. Med. 3, 197–204. 10.1142/S0192415X81000263 7053019

[B3] Chi L. Wang J. B. Chen T. Sun C. Wei L. Y. (2022). Textual research on efficacy of niter based on Ancient medical books. Mod. Chin. Med. 24, 2483–2488. 10.13313/j.issn.1673-4890.20220422001

[B4] Chi L. Chen T. Wang J. B. (2025). Historical application of mineral drugs based on the classified case records of famous physicians: a case Study of gyosum fibrosum. World Chin. Med. 10.3969/j.issn.1673-7202.2024.24.011

[B5] Fujikawa Y. (1944). A history of diseases in Japan. Tokyo: Nihon Isho Shuppan Co., Ltd.

[B6] Fukae S. (2024). Honzouwamyou. asakusashinteramachi(edo): izumiyashōjirō. (918).

[B7] Heinrich M. Jalil B. Andel-Tawab M. Echeverria J. Kulic Z. McGaw L. J. (2022). Best Practice in the chemical characterisation of extracts used in pharmacological and toxicological research—The ConPhyMP—Guidelines. Front. Pharmacol. 13, 01–20. 10.3389/fphar.2022.953205 PMC9514875 36176427

[B8] Hosseinkhani A. Hosamo A. Montaseri H. Mehdizadeh A. Zomorodian K. Zarshenas M. M. (2025). Physicochemical, pharmaceutical and toxicological evaluation of Armenian bole, an Ancient medicinal clay. Pharm. Chem. J. 58 (11), 1669–1678. 10.1007/s11094-025-03336-w

[B9] Huan Y. Qin S. Yue L. Nan S. Zuo T. Miao M. (2024). Dosing characteristics of mineral medicines listed in Chinese Materia Medica. Chin. J. Hosp. Pharm. 44 (17), 2044–2061. 10.13286/j.1001-5213.2024.17.14

[B10] Ihara N. (1901). *Teikoku Bussan Chishi* (Imperial monograph of local products). Tokyo: Shunyodo.

[B11] Kosoto H. (1999). Nihon kanpō tenseki jiten. Tokyo: Taishukan Publishing Co., Ltd.

[B12] Lin L. Liu G. Z. Ren X. Y. Zhang L. Tan L. J. Yu F. R. (2025). Research progress and thinking of mineral Chinese medicine. Chin. Pharm. J. Available online at: https://link.cnki.net/urlid/11.2162.R.20250327.1154.002 (Accessed May 3, 2025).

[B13] Liu W. X. Zhou G. Li G. Min D. (2011). Research of origin and ethnopharmacological uses of mineral medicine *Halitum* . China J. Chin. Materia Medica 36, 2445–2449. 10.4268/cjcmm20111730 22121821

[B14] Liu S. J. Wu S. C. Ma Y. L. Zhao Q. Ao W. Wang X. X. (2023). Investigation and analysis of mineral Chinese medicine species, market circulation and clinical application in China. Chin. Traditional Herb. Drugs 19, 6555–6568. 10.7501/j.issn.0253-2670.2023.19.035

[B15] Ma J. X. (1990). TCM literature. Shanghai: Shanghai Scientific and Technical Publishers, 42–43.

[B16] Makbul S. A. A. Jahan N. Ahmad G. (2018). Hajrul yahood (*Lapis judaicus*): an important mineral drug of Unani system of medicine for the management of urolithiasis. J. Ethnopharmacol. 222, 165–170. 10.1016/j.jep.2018.04.047 29733943

[B17] Masutomi K. (1957). Research on Ancient mineral medicines with a focus on the medicines stored in shosoin. Jpn. Soc. Pharmacogn. 11 (2), 17–19.

[B18] Masutomi K. Yamasaki K. On the Lu-Kan-Shih (1953). Jpn. J. Pharmacogn. 6 (1), 23–24.

[B19] Mayanagi M. (2014). Medical history of the Chinese character cultural sphere. Fuzhou: Fujian Science and Technology Publishing House.

[B20] Ministry of Health, Labour and Welfare of Japan (2021). The Japanese pharmacopoeia. 18th edition. Available online at: https://www.mhlw.go.jp/content/11120000/000788459.pdf (Accessed May 5, 2025).

[B21] Monta S. (2020). The historical appearance of *limonite* as a stone medicine: also an examination of the theory of its existence in the yayoi period. J. Jpn. religious Cult. Hist. 24, 1–15.

[B22] National Administration of Traditional Chinese Medicine (1999). Chinese Materia Medica. Shanghai: Shanghai Scientific and Technical Publishers.

[B23] National Medical Products Administration of China (2025). Pharmacopoeia of the People'S Republic of China. Beijing: China Medical Science Press.

[B24] Nghi H. T. Shahmohammadi S. Ebrahimi K. H. (2023). Ancient complexes of iron and sulfur modulate oncogenes and oncometabolism. Curr. Opin. Chem. Biologu 76, 102338–8. 10.1016/j.cbpa.2023.102338 37295349

[B25] Okamoto Y. (1944). *Fukuoka-ken Kobutsu-shi* (A monograph of the minerals of Fukuoka prefecture). Tokyo: Nihon Kobutsu Shumi no Kai Shuppanbu.

[B26] Onda S. (1901). An overview of Japanese natural products. Tetsukakubō.

[B27] Sikder M. M. (2024). Ayurvedic medicine: a traditional medical System and its heavy metal poisoning. Chonnam Med. J. 60 (2), 97–104. 10.4068/cmj.2024.60.2.97 38841605 PMC11148304

[B28] Su G. Chen G. An X. Wang H. Pei Y.-H. (2017). Metabolic profiling analysis of the alleviation effect of treatment with baicalin on cinnabar induced toxicity in rats urine and serum. Front. Pharmacol. 8, 271. 10.3389/fphar.2017.00271 28567014 PMC5434134

[B29] Su H. X. Li R. X. Zhang M. Ma B. X. (2025). Analysis of QIAN Yi’s experience in using mineral Chinese medicine from *Xiaoer Yaozheng Zhijue* . Guid. J. Traditional Chin. Med. Pharm. 31, 221–225. 10.13862/j.cn43-1446/r.2025.04.042

[B30] Sugiyama H. (1997). On the stone medicines among the treasures of the shosoin and the investigation conducted by Mr. Masutomi kazunosuke. J. kampo Med. 44 (7), 885–895.

[B31] Tachiki O. (1989). Apropos of the wooden tablets on medicine enearthed from the ruins of fujiwara palace and an aspect of Ancient medical care — on using of mineral drugs during 7th-8th centuries. Cult. antiqu 41 (12), 44–54.

[B32] Tamba Y. (2024). Ishinhou. Hanai family manuscript version. (984).

[B33] Tanaka Y. (1901). “ *Nihon bussan nenpyō* (chronological table of Japanese products),” in Tokyo: jūmonji shōkai.

[B34] Tanaka H. (1988). *Kingin-to nihon* (the gold and silver Island Japan). Tokyo: Kobundo.

[B35] Tanaka K. (2020). Shōyaku to kanpōyaku no jiten [Japanese]. Tokyo: nihon Bungeisha, 129.

[B36] Tian H. Zhang R. Jiang H. F. Yan H. Yang Y. C. (2024). Textual research on Chinese mineral drug *Succinum* . Chin. J. Ethnomedicine Ethnopharmacy 33, 40–47. 10.3969/j.issn.1007-8517.2024.19.zgmzmjyyzz202419009

[B37] Tokoi H. Saito T. (1979). *Nihon Sanbutsu-shi* (A monograph on Japanese products) (Tokyo: Yasaka Shobo).

[B38] Wada A. (1999). The ingestion of stone medicines in Ancient Japan. Anc. Cult. East Asia 11, 276–277.

[B39] Wang X. T. (2007). A definitive work on the standardization of traditional Chinese medicine names — *research on the verification and standardization of traditional Chinese medicine names* . China J. Chin. Materia Medica 32 (12), 2211–2212.

[B40] Wang D. Zong C. (2024). Construction and disclosure of “Dadi Corpus”. J. Japnese Lang. Study Res. 2, 125–127. 10.13508/j.cnki.jsr.2024.02.007

[B41] Watanabe S. (2024). Wasan Sekiyaku Chimeiki [Records of place names of Japanese-produced stone medicines, manuscript]. Tokyo (Japan): held in national Diet Library, 1889. Japanese.

[B42] Xiang R. X. Zhang H. M. (2021). Effect of cultural exchange on traditional Chinese medicine in Tang dynasty — taking *shi Yao Er Ya* mineral medicine as an example. J. Chengdu Univ. Traditional Chin. Med. 44, 109–112. 10.13593/j.cnki.51-1501/r.2021.04.109

[B43] Xiang J. Y. Chi Y. Y. Han J. X. Xiang H. Y. Xie Q. H. (2021). The toxicity and attenuation methods of toxic Chinese materia Medica for its reasonable application: a review. Am. J. Chin. Med. 49 (1), 41–67. 10.1142/S0192415X21500038 33416023

[B44] Yan X. Y. Chen Y. Y. Liang X. T. Cai Q. Z. Huang Z. H. (2022). Characteristics of drugs in the jade section of the book of book of *Bencao Tujing* from data analysis. Strait Pharm. J. 34, 30–35.

[B45] Youno C. Martin J. Fleurentin J. Mazars G. Nottera D. Mortier F. (1991). Repertory, therapeutic indications, chemical analysis and cultural background of mineral drugs of Afghanistan. J. Ethnopharmacol. 33, 169–178. 10.1016/0378-8741(91)90175-d 1943165

[B46] Zhao M. Li Y. Wang Z. (2022). Mercury and mercury-containing preparations: history of use, clinical applications, pharmacology, toxicology, and pharmacokinetics in traditional Chinese medicine. Front. Pharmacol. 13, 807807. 10.3389/fphar.2022.807807 35308204 PMC8924441

